# Supplementary material for: Using electronic health records to assess the relationship between colonization pressure and nosocomial pathogen acquisition
Source: Nat Commun. 2026 Feb 24;17:3134. doi: 10.1038/s41467-026-69873-4 (PMC13043742; doi:10.1038/s41467-026-69873-4)
Supplement: Supplementary file 1 — Supplementary Information [file 41467_2026_69873_MOESM1_ESM.pdf]

SUPPLEMENTARY MATERIAL

Table of contents

	Description	Page
Information		
S1	STROBE / RECORD Checklist	3
Tables		
S1	Antibiotic categories	7
S2	Flow diagrams for all cohorts	8
S3	Baseline characteristics for *E. coli* and ESBL *E. coli* cohorts.	9
S4	Baseline characteristics for *K. pneumoniae*, and ESBL *K. pneumoniae* cohorts.	10
S5	Baseline characteristics for vancomycin susceptible *E. faecalis*, vancomycin resistant *E. faecium*, and *C. difficile* cohorts.	11
S6	Baseline characteristics for MSSA and MRSA cohorts.	12
S7	Baseline cohort characteristics for drug susceptible *P. aeruginosa*, and drug resistant *P. aeruginosa* cohorts	13
S8	Exponentiated coefficients for conditional logistic regression models	14
S9	Performance of conditional logistic regression and XGBoost models	15
S10	Mean SHAP values for XGB models	16
Figures		
S1	Distribution of individual Elixhauser categories for drug susceptible and ESBL *E. coli* and *K. pneumoniae*	17
S2	Distribution of individual Elixhauser categories for *C. difficile*, vancomycin susceptible *E. faecalis* and vancomycin resistant *E. faecium*	18
S3	Distribution of individual Elixhauser categories for MSSA, MRSA, drug susceptible and drug resistant *P. aeruginosa*	19
S4	Number of antibiotic courses per 100 people in the previous 60 days by class	20
S5	Cumulative distributions of colonization pressure	21
S6	Number of ward co-occupants with drug susceptible and ESBL Enterobacterales	22
S7	Number of ward co-occupants with *C. difficile*	23
S8	Number of ward co-occupants with vancomycin susceptible and vancomycin resistant *Enterococcus* species	24
S9	Number of ward co-occupants with MSSA and MRSA	25
S10	Number of ward co-occupants with drug susceptible and drug resistant *P. aeruginosa*	26

S11	SHAP values from XGB models for drug susceptible and ESBL *E. coli*	27
S12	SHAP values from XGB models for drug susceptible and ESBL *K. pneumoniae*	28
S13	SHAP values from XGB models for *C. difficile*	29
S14	SHAP values from XGB models for vancomycin susceptible *E. faecalis* and vancomycin resistant *E. faecium*	30
S15	SHAP values from XGB models for MSSA and MRSA	31
S16	SHAP values from XGB models for drug susceptible and drug resistant *P. aeruginosa*	32

	Item No.	STROBE items	Location in manuscript where items are reported	RECORD items	Location in manuscript where items are reported
Title and abstract					
	1	(a) Indicate the study's design with a commonly used term in the title or the abstract (b) Provide in the abstract an informative and balanced summary of what was done and what was found	(a) Abstract - Line 26-32<br><br>(b) Abstract - Line 21-41	RECORD 1.1: The type of data used should be specified in the title or abstract. When possible, the name of the databases used should be included.<br><br>RECORD 1.2: If applicable, the geographic region and timeframe within which the study took place should be reported in the title or abstract.<br><br>RECORD 1.3: If linkage between databases was conducted for the study, this should be clearly stated in the title or abstract.	RECORD 1.1: Abstract - Line 26-30<br><br>RECORD 1.2: Abstract - Line 26-30<br><br>RECORD 1.3: Not Applicable
Introduction					
Background rationale	2	Explain the scientific background and rationale for the investigation being reported	Introduction - Line 43-65		
Objectives	3	State specific objectives, including any prespecified hypotheses	Introduction - Line 65-71		
Methods					
Study Design	4	Present key elements of study design early in the paper	Methods - Study design - Line 314-319		
Setting	5	Describe the setting, locations, and relevant dates, including periods of recruitment, exposure, follow-up, and data collection	Methods - Study design - 314-319		
Participants	6	(a) Cohort study - Give the eligibility criteria, and the sources and methods of selection of participants. Describe methods of follow-up<br>Case-control study - Give the eligibility criteria, and the sources and methods of case ascertainment and control selection. Give the rationale for the choice of cases and controls<br>Cross-sectional study - Give the eligibility criteria, and the sources and methods of selection of participants<br><br>(b) Cohort study - For matched studies, give matching criteria and number of exposed and unexposed	(a) Methods - Cohort definition - Line 320-343<br><br>(b) Methods - Cohort definition - Line 320-343	RECORD 6.1: The methods of study population selection (such as codes or algorithms used to identify subjects) should be listed in detail. If this is not possible, an explanation should be provided.<br><br>RECORD 6.2: Any validation studies of the codes or algorithms used to select the population should be referenced. If validation was conducted for this study and not published elsewhere, detailed methods and results should be provided.<br><br>RECORD 6.3: If the study involved linkage of databases, consider use of a flow diagram or other graphical display to demonstrate the data linkage process,	RECORD 6.1: Data Availability - Line 401-408, Code Availability - Line 409-411, GitHub Repository (link provided).<br><br>RECORD 6.2: Methods - Statistical analysis - Line 379-400, Code Availability - Line 409 - 411, Physionet

		Case-control study - For matched studies, give matching criteria and the number of controls per case		including the number of individuals with linked data at each stage.	RECORD 6.3: Not Applicable
Variables	7	Clearly define all outcomes, exposures, predictors, potential confounders, and effect modifiers. Give diagnostic criteria, if applicable.	Methods - Model covariates - Line 358-378	RECORD 7.1: A complete list of codes and algorithms used to classify exposures, outcomes, confounders, and effect modifiers should be provided. If these cannot be reported, an explanation should be provided.	RECORD 7.1: Code Availability – Line 409 – 411, GitHub Repository (Link provided).
Data sources/ measurement	8	For each variable of interest, give sources of data and details of methods of assessment (measurement).<br>Describe comparability of assessment methods if there is more than one group	Methods - Study design Line 314-319, Methods - Model covariates 358-378		
Bias	9	Describe any efforts to address potential sources of bias	Methods - Cohort definition - Line 320-343, Methods - Model covariates - Line 358-378		
Study size	10	Explain how the study size was arrived at	Methods - Cohort definition - Line 320-343, Supplementary Table S2		
Quantitative variables	11	Explain how quantitative variables were handled in the analyses. If applicable, describe which groupings were chosen, and why	Methods - Model covariates - Line 358-378		
Statistical methods	12	(a) Describe all statistical methods, including those used to control for confounding<br>(b) Describe any methods used to examine subgroups and interactions<br>(c) Explain how missing data were addressed<br>(d) Cohort study - If applicable, explain how loss to follow-up was addressed<br>Case-control study - If applicable, explain how matching of cases and controls was addressed<br>Cross-sectional study - If applicable, describe analytical methods taking account of sampling strategy<br>(e) Describe any sensitivity analyses	(a) Methods - Statistical analyses, Line 379 - 400<br>(b) Not Applicable<br>(c) Data Availability - Line 401-408, Code Availability - Line 409-411, Physionet (Link Provided)<br>(d) Methods - Cohort Definition - Line 320-343<br>(e) Not Applicable		
Data access and cleaning methods		..		RECORD 12.1: Authors should describe the extent to which the investigators had access to the database population used to create the study population.	RECORD 12.1: Data Availability - Line 401-408,

				RECORD 12.2: Authors should provide information on the data cleaning methods used in the study.	Code Availability - Line 409-411<br><br>RECORD 12.2: Physionet (Link Provided), Github Repository (Link Provided)
Linkage		..		RECORD 12.3: State whether the study included person-level, institutional-level, or other data linkage across two or more databases. The methods of linkage and methods of linkage quality evaluation should be provided.	Method - Study design - Line 314-319
Results					
Participants	13	(a) Report the numbers of individuals at each stage of the study (e.g., numbers potentially eligible, examined for eligibility, confirmed eligible, included in the study, completing follow-up, and analysed)<br>(b) Give reasons for non-participation at each stage.<br>(c) Consider use of a flow diagram	Supplementary Table S2	RECORD 13.1: Describe in detail the selection of the persons included in the study (i.e., study population selection) including filtering based on data quality, data availability and linkage. The selection of included persons can be described in the text and/or by means of the study flow diagram.	Method - Cohort definition - Line 320-343, Supplementary Table S2
Descriptive data	14	(a) Give characteristics of study participants (e.g., demographic, clinical, social) and information on exposures and potential confounders<br>(b) Indicate the number of participants with missing data for each variable of interest<br>(c) Cohort study - summarise follow-up time (e.g., average and total amount)	(a) Results - Line 73-96, Figure 3<br>(b) Physionet (Link Provided)		
Outcome data	15	Cohort study - Report numbers of outcome events or summary measures over time<br>Case-control study - Report numbers in each exposure category, or summary measures of exposure<br>Cross-sectional study - Report numbers of outcome events or summary measures	Results - Line 73-96		
Main results	16	(a) Give unadjusted estimates and, if applicable, confounder-adjusted estimates and their precision (e.g., 95% confidence interval). Make clear which confounders were adjusted for and why they were included<br>(b) Report category boundaries when continuous variables were categorized<br>(c) If relevant, consider translating estimates of relative risk into absolute risk for a meaningful time period	(a) Results - Line 97-160<br>(b) Not Applicable<br>(c) Not Applicable		

Other analyses	17	Report other analyses done—e.g., analyses of subgroups and interactions, and sensitivity analyses	Results - Line 161-171		
Discussion					
Key results	18	Summarise key results with reference to study objectives	Discussion - Line 173-181		
Limitations	19	Discuss limitations of the study, taking into account sources of potential bias or imprecision. Discuss both direction and magnitude of any potential bias	Discussion - Line 275-301	RECORD 19.1: Discuss the implications of using data that were not created or collected to answer the specific research question(s). Include discussion of misclassification bias, unmeasured confounding, missing data, and changing eligibility over time, as they pertain to the study being reported.	Discussion - Line 287-301
Interpretation	20	Give a cautious overall interpretation of results considering objectives, limitations, multiplicity of analyses, results from similar studies, and other relevant evidence	Discussion - Line 302-310		
Generalisability	21	Discuss the generalisability (external validity) of the study results	Discussion - Line 252-266, Line 275-286		
Other Information					
Funding	22	Give the source of funding and the role of the funders for the present study and, if applicable, for the original study on which the present article is based	Acknowledgement - Line 516-521		
Accessibility of protocol, raw data, and programming code		..		RECORD 22.1: Authors should provide information on how to access any supplemental information such as the study protocol, raw data, or programming code.	Data Availability - Line 401-408, Code Availability - Line 409-411

Supplementary information S1: STROBE / RECORD checklist.

*Reference: Benchimol EI, Smeeth L, Guttmann A, Harron K, Moher D, Petersen I, Sørensen HT, von Elm E, Langan SM, the RECORD Working Committee. The REporting of studies Conducted using Observational Routinely-collected health Data (RECORD) Statement. PLoS Medicine 2015.

*Checklist is protected under Creative Commons Attribution (CC BY) license.

Drug class	Sub-class	Group	Antibiotic
Anti-anaerobe		1	Metronidazole
β-lactams	Penicillins	2	Penicillin
			Amoxicillin
			Ampicillin
	Extended spectrum penicillins	3	Amoxicillin-clavulanate
			Ampicillin-sulbactam
			Piperacillin-tazobactam
	Anti-staphylococcal penicillins	4	Dicloxacillin
			Nafcillin
			Oxacillin
			Cefazolin
	Cephalosporins	5	Cephalexin
			Cefadroxil
			Cefaclor
			Cefuroxime
			Cefprozil
			Cefpodoxime
			Cefdinir
			Cefotetan
			Cefixime
			Cefoxitin
	Extended spectrum cephalosporins	6	Cefotaxime
			Ceftazidime
			Ceftriaxone
			Cefepime
			Ceftaroline
			Cefiderocol
	Carbapenems	7	Ertapenem
			Imipenem
			Meropenem
Fluoroquinolones		8	Moxifloxacin
			Ciprofloxacin
			Levofloxacin
			Delafloxacin
Folate inhibitors		9	Sulfamethoxazole
			Trimethoprim
			Trimethoprim-sulfamethoxazole
Glycopeptides	Systemic	10	Vancomycin
	Anti-*C. difficile*	11	Vancomycin (oral)
Lincosamides		12	Clindamycin
Macrolides		13	Clarithromycin
			Azithromycin
Tetracyclines		14	Tetracycline
			Demeclocycline
			Doxycycline
			Minocycline
			Tigecycline
			Omadacycline
			Eravacycline

Table S1: Antibiotic categories

Pathogen	Total admitted patients with pathogen of interest	No antibiotic treatment between -7 days to +3 days	No prior encounters within 90 days	Pathogens of interest identified after day 3 and before day 30	Drop cases not in the first room	Drop cases with prior infection	Controls matched to cases
E. coli	64,492	44,653	30,344	7,594	6,137	5,701	3,585
ESBL *E. coli*	16,482	10,462	5,890	1,783	1,323	1,203	996
K. pneumoniae	26,848	17,627	10,245	2,789	2,165	2,037	1,704
ESBL *K. pneumoniae*	6,662	4,033	1,813	639	437	404	238
VS *E. faecalis*	34,875	22,763	13,209	4,030	3,031	2,864	1,823
VR *E. faecium*	8,461	2,119	2,054	837	521	482	244
C. difficile	13,053	8,659	4,342	1,883	1,415	1,365	592
MSSA	41,275	26,441	16,972	4,593	3,573	3,369	2,952
MRSA	21,503	12,843	7,225	1,897	1,443	1,305	1,101
DS *P. aeruginosa*	21,257	12,664	7,115	2,427	1,812	1,694	1,309
DR *P. aeruginosa*	9,312	5,373	2,729	832	627	561	379

Table S2: Flow diagrams for all cohort cases. Abbreviations, VS, vancomycin susceptible; VR, vancomycin resistant; DS, drug susceptible; DR, drug resistant

Feature	*E. coli*		ESBL *E. coli*	
	Case	Control	Case	Control
Sample size	3,585	5,693	996	2,299
Demographics				
Age (mean, SE)	66.5 +/- 0.3	66.7 +/- 0.3	66.7 +/- 0.6	68.3 +/- 0.4
Gender (% female)	71.1	69.6	61.7	63.0
Elixhauser index (mean, SE)	9.7 +/- 0.3	9.3 +/- 0.2	10.8 +/- 0.5	9.6 +/- 0.3
Prior surgery (%)	13.0	10.8	15.9	13.0
Length of Stay (median, IQR)	4.5 (2.9, 7.9)	3.8 (2.8, 5.0)	5.1 (3.5, 8.5)	3.9 (2.9, 5.7)
Matching Duration (median, IQR)	3.9 (2.9, 5.6)	3.8 (2.8, 5.0)	4.2 (3.2, 6.1)	3.9 (2.9, 5.7)
Time to Infection (median, IQR)	9.0 (4.9, 17.2)	0.0 (0.0, 0.0)	8.8 (5.1, 17.6)	0.0 (0.0, 0.0)
Prior antimicrobials (%)				
β-lactams				
Penicillins	0.2	0.2	0.4	0.4
Extended spectrum penicillins	0.6	0.5	0.9	0.5
Cephalosporins	0.8	0.6	1.4	0.9
Extended spectrum cephalosporins	0.0	0.0	0.0	0.0
Anti-Staphylococcal β-lactams	0.1	0.1	0.1	0.0
Other cell wall active agents				
Glycopeptides	0.0	0.0	0.0	0.0
DNA synthesis inhibitors				
Sulfonamides	0.7	0.5	1.3	1.0
Protein synthesis inhibitors				
Fluoroquinolones	0.6	0.4	1.8	1.4
Tetracyclines	0.4	0.4	0.4	0.2
Macrolides	0.2	0.2	0.1	0.1
Lincosamides	0.1	0.1	0.0	0.0
Anti-anaerobic antibiotics				
Metronidazole	0.2	0.1	0.4	0.3
Oral vancomycin	0.0	0.0	0.0	0.0

Table S3: Baseline characteristics for *E. coli*, ESBL *E. coli* cohorts.

Abbreviations, SE, standard error; IQR, Interquartile Range.

Feature	*K. pneumoniae*		ESBL *K. pneumoniae*	
	Case	Control	Case	Control
Sample size	1,704	3,684	238	376
Demographics				
Age (mean, SE)	67.8 +/- 0.4	68.4 +/- 0.3	69.3 +/- 1.0	69.0 +/- 0.9
Gender (% female)	58.2	60.4	50.4	53.5
Elixhauser index (mean, SE)	11.6 +/- 0.4	9.4 +/- 0.3	12.9 +/- 1.0	10.2 +/- 0.8
Prior surgery (%)	14.4	13.6	8.8	8.1
Length of Stay (median, IQR)	5.3 (3.5, 8.9)	4.0 (3.0, 5.6)	6.3 (3.7, 12.1)	4.8 (3.2, 7.9)
Matching Duration (median, IQR)	4.5 (3.2, 6.0)	4.0 (3.0, 5.6)	4.9 (3.5, 8.0)	4.8 (3.2, 7.9)
Time to Infection (median, IQR)	8.8 (5.2, 17.1)	0.0 (0.0, 0.0)	11.4 (6.1, 19.0)	0.0 (0.0, 0.0)
Prior antimicrobials (%)				
β-lactams				
Penicillins	0.4	0.3	0.0	0.0
Extended spectrum penicillins	0.5	0.4	0.0	0.0
Cephalosporins	1.3	1.1	0.8	0.8
Extended spectrum cephalosporins	0.1	0.0	0.0	0.0
Anti-Staphylococcal β-lactams	0.0	0.0	0.0	0.0
Other cell wall active agents				
Glycopeptides	0.1	0.0	0.0	0.0
DNA synthesis inhibitors				
Sulfonamides	0.8	0.7	2.1	1.6
Protein synthesis inhibitors				
Fluoroquinolones	1.5	1.2	1.7	1.6
Tetracyclines	0.8	0.7	0.4	0.5
Macrolides	0.6	0.4	0.0	0.0
Lincosamides	0.2	0.1	0.0	0.0
Anti-anaerobic antibiotics				
Metronidazole	0.1	0.1	0.0	0.0
Oral vancomycin	0.1	0.1	0.0	0.0

Table S4: Baseline characteristics for *K. pneumoniae*, and ESBL *K. pneumoniae* cohorts. Abbreviations, SE, standard error; IQR, Interquartile Range.

Feature	Vancomycin susceptible *E. faecalis*		Vancomycin resistant *E. faecium*		*C. difficile*	
	Case	Control	Case	Control	Case	Control
Sample size	1,823	3,082	244	366	592	881
Demographics						
Age (mean, SE)	65.7 +/- 0.5	65.8 +/- 0.4	70.7 +/- 0.9	70.7 +/- 0.8	68.9 +/- 0.6	70.7 +/- 0.5
Gender (% female)	53.3	52.8	59.0	60.9	56.4	58.4
Elixhauser index (mean, SE)	10.3 +/- 0.4	9.6 +/- 0.3	14.3 +/- 1.0	12.0 +/- 0.8	13.4 +/- 0.7	9.9 +/- 0.5
Prior surgery (%)	17.9	17.7	13.9	11.7	14.7	12.9
Length of Stay (median, IQR)	4.6 (3.0, 7.9)	3.8 (2.8, 5.0)	7.3 (4.5, 13.7)	4.8 (3.8, 7.6)	5.8 (3.2, 11.0)	4.0 (2.9, 6.0)
Matching Duration (median, IQR)	4.0 (3.0, 5.8)	3.8 (2.8, 5.0)	5.7 (3.9, 8.9)	4.8 (3.8, 7.6)	4.1 (3.1, 7.0)	4.0 (2.9, 6.0)
Time to Infection (median, IQR)	9.9 (5.4, 18.0)	0.0 (0.0, 0.0)	10.5 (6.0, 17.6)	0.0 (0.0, 0.0)	9.0 (5.0, 16.8)	0.0 (0.0, 0.0)
Prior antimicrobials (%)						
β-lactams						
Penicillins	0.3	0.3	0.4	0.3	0.2	0.1
Extended spectrum penicillins	0.8	0.6	0.0	0.0	0.8	0.7
Cephalosporins	1.3	1.2	2.5	2.2	2.7	2.4
Extended spectrum cephalosporins	0.0	0.0	0.0	0.0	0.0	0.0
Anti-Staphylococcal β-lactams	0.0	0.0	0.4	0.3	0.0	0.0
Other cell wall active agents						
Glycopeptides	0.0	0.0	0.0	0.0	0.0	0.0
DNA synthesis inhibitors						
Sulfonamides	1.2	0.9	1.2	1.4	0.8	0.6
Protein synthesis inhibitors						
Fluoroquinolones	1.4	1.3	1.2	1.1	3.0	2.7
Tetracyclines	0.5	0.6	0.4	0.3	0.8	0.8
Macrolides	0.4	0.4	0.4	0.5	0.3	0.3
Lincosamides	0.1	0.0	0.0	0.0	0.0	0.0
Anti-anaerobic antibiotics						
Metronidazole	0.1	0.0	0.0	0.0	1.2	1.1
Oral vancomycin	0.0	0.0	0.4	0.5	0.2	0.1

Table S5: Baseline characteristics for vancomycin susceptible *E. faecalis*, vancomycin resistant *E. faecium*, and *C. difficile* cohorts.

Abbreviations, SE, standard error; IQR, Interquartile Range.

Feature	MSSA		MRSA	
	Case	Control	Case	Control
Sample size	2,952	6,504	1,101	2,397
Demographics				
Age (mean, SE)	52.1 +/- 0.5	54.7 +/- 0.3	58.9 +/- 0.7	57.7 +/- 0.5
Gender (% female)	44.6	47.4	44.0	45.5
Elixhauser index (mean, SE)	8.8 +/- 0.3	7.3 +/- 0.2	9.0 +/- 0.5	8.1 +/- 0.3
Prior surgery (%)	15.4	15.4	11.3	11.5
Length of Stay (median, IQR)	5.7 (3.5, 10.1)	3.9 (2.9, 5.4)	5.8 (3.6, 9.8)	4.0 (2.9, 5.7)
Matching Duration (median, IQR)	4.2 (3.2, 5.9)	3.9 (2.9, 5.4)	4.4 (3.2, 6.0)	4.0 (2.9, 5.7)
Time to Infection (median, IQR)	7.9 (4.8, 16.1)	0.0 (0.0, 0.0)	8.0 (4.9, 16.6)	0.0 (0.0, 0.0)
Prior antimicrobials (%)				
β-lactams				
Penicillins	0.2	0.2	0.4	0.4
Extended spectrum penicillins	0.6	0.6	1.0	0.8
Cephalosporins	0.8	0.8	1.5	1.6
Extended spectrum cephalosporins	0.0	0.0	0.0	0.0
Anti-Staphylococcal β-lactams	0.0	0.0	0.0	0.0
Other cell wall active agents				
Glycopeptides	0.0	0.0	0.0	0.0
DNA synthesis inhibitors				
Sulfonamides	0.4	0.3	0.5	0.4
Protein synthesis inhibitors				
Fluoroquinolones	0.5	0.4	1.5	1.1
Tetracyclines	0.5	0.3	0.6	0.6
Macrolides	0.2	0.2	0.8	0.8
Lincosamides	0.1	0.1	0.1	0.0
Anti-anaerobic antibiotics				
Metronidazole	0.0	0.0	0.1	0.0
Oral vancomycin	0.0	0.0	0.1	0.0

Table S6: Baseline characteristics for MSSA and MRSA cohorts.

Abbreviations, SE, standard error; IQR, Interquartile Range.

Feature	Drug susceptible *P. aeruginosa*		Drug resistant *P. aeruginosa*	
	Case	Control	Case	Control
Sample size	1,309	2,533	379	665
Demographics				
Age (mean, SE)	68.3 +/- 0.5	68.4 +/- 0.4	69.3 +/- 0.8	69.6 +/- 0.6
Gender (% female)	49.7	51.4	44.1	47.3
Elixhauser index (mean, SE)	11.3 +/- 0.4	9.5 +/- 0.3	12.1+/- 0.8	10.7 +/- 0.6
Prior surgery (%)	16.6	16.7	12.7	11.9
Length of Stay (median, IQR)	5.2 (3.7, 8.9)	4.0 (2.9, 5.6)	5.9 (3.7,12.1)	4.0 (3.0, 5.8)
Matching Duration (median, IQR)	4.4 (3.4, 5.8)	4.0 (2.9, 5.6)	4.5 (3.3, 6.8)	4.0 (3.0, 5.8)
Time to Infection (median, IQR)	8.3 (5.0, 17.0)	0.0 (0.0,0.0)	9.8 (5.1, 17.4)	0.0 (0.0, 0.0)
Prior antimicrobials (%)				
β-lactams				
Penicillins	0.1	0.1	0.3	0.3
Extended spectrum penicillins	1.0	0.9	0.3	0.3
Cephalosporins	2.1	2.4	1.3	0.9
Extended spectrum cephalosporins	0.2	0.2	0.0	0.0
Anti-Staphylococcal β-lactams	0.0	0.0	0.0	0.0
Other cell wall active agents				
Glycopeptides	0.0	0.0	0.0	0.0
DNA synthesis inhibitors				
Sulfonamides	1.0	0.9	1.6	1.1
Protein synthesis inhibitors				
Fluoroquinolones	1.3	1.3	1.1	1.4
Tetracyclines	0.8	0.9	1.1	1.2
Macrolides	1.1	1.3	1.6	1.1
Lincosamides	0.2	0.2	0.3	0.2
Anti-anaerobic antibiotics				
Metronidazole	0.0	0.0	0.0	0.0
Oral vancomycin	0.1	0.0	0.0	0.0

Table S7: Baseline cohort characteristics for drug susceptible *P. aeruginosa*, and drug resistant *P. aeruginosa* cohorts

Abbreviations, SE, standard error; IQR, Interquartile Range.

Model feature	Target pathogen			
	Gram negative enteric flora			
	E. coli	**ESBL *E. coli***	***K. pneumoniae***	**ESBL *K. pneumoniae***
Colonization pressure				
DS Enterobacterales	**1.01 (1.00, 1.02)**	**0.98 (0.96, 0.99)**	0.99 (0.98, 1.00)	**0.96 (0.93, 1.00)**
ESBL Enterobacterales	**0.96 (0.94, 0.98)**	1.03 (1.00, 1.07)	0.99 (0.96, 1.02)	**1.10 (1.03, 1.19)**
C. difficile	**0.96 (0.93, 0.99)**	0.96 (0.90, 1.01)	**0.95 (0.91, 0.99)**	0.88 (0.77, 1.01)
VSE	1.01 (0.99, 1.03)	**1.05 (1.01, 1.09)**	**1.03 (1.00, 1.06)**	0.98 (0.91, 1.07)
VRE	1.03 (1.00, 1.07)	1.00 (0.94, 1.07)	1.04 (0.99, 1.10)	**1.29 (1.11, 1.51)**
MSSA	1.01 (0.99, 1.03)	1.02 (0.98, 1.06)	1.00 (0.97, 1.02)	0.92 (0.85, 1.00)
MRSA	**0.95 (0.92, 0.98)**	0.98 (0.92, 1.03)	**0.95 (0.91, 0.99)**	0.99 (0.87, 1.12)
DS *P. aeruginosa*	1.00 (0.97, 1.03)	1.00 (0.95, 1.05)	1.01 (0.97, 1.05)	1.01 (0.89, 1.13)
DR *P. aeruginosa*	0.98 (0.94, 1.03)	1.01 (0.94, 1.08)	1.05 (0.99, 1.11)	1.01 (0.86, 1.18)
Elixhauser index	1.00 (1.00, 1.01)	**1.01 (1.00, 1.01)**	**1.01 (1.00, 1.01)**	1.01 (1.00, 1.02)
	Gram positive enteric flora			
	C. difficile	**VS *E. faecalis***	**VR *E. faecium***	
Colonization pressure				
DS Enterobacterales	**0.98 (0.96, 1.00)**	**0.98 (0.97, 0.99)**	**0.96 (0.93, 0.99)**	
ESBL Enterobacterales	0.98 (0.93, 1.03)	1.01 (0.98, 1.04)	0.94 (0.86, 1.02)	
C. difficile	**1.33 (1.22, 1.44)**	**0.91 (0.86, 0.95)**	1.13 (1.00, 1.28)	
VSE	**1.06 (1.00, 1.12)**	**1.12 (1.09, 1.15)**	**1.09 (1.01, 1.17)**	
VRE	0.99 (0.90, 1.09)	1.05 (1.00, 1.10)	1.09 (0.96, 1.23)	
MSSA	0.97 (0.92, 1.02)	0.99 (0.96, 1.02)	0.97 (0.90, 1.05)	
MRSA	0.94 (0.86, 1.03)	0.97 (0.92, 1.01)	1.09 (0.96, 1.22)	
DS *P. aeruginosa*	0.93 (0.86, 1.01)	1.01 (0.96, 1.05)	1.00 (0.90, 1.12)	
DR *P. aeruginosa*	0.94 (0.84, 1.04)	**0.90 (0.84, 0.96)**	0.91 (0.78, 1.06)	
Elixhauser index	**1.01 (1.00, 1.02)**	1.00 (1.00, 1.01)	1.01 (0.94, 1.02)	
	Skin flora			
	**MSSA**	**MRSA**		
Colonization pressure				
DS Enterobacterales	**0.96 (0.95, 0.97)**	**0.98 (0.96, 0.99)**		
ESBL Enterobacterales	**0.97 (0.94, 0.99)**	0.97 (0.94, 1.00)		
C. difficile	**0.94 (0.90, 0.98)**	0.99 (0.93, 1.04)		
VSE	0.99 (0.97, 1.02)	0.99 (0.95, 1.03)		
VRE	**1.07 (1.03, 1.11)**	1.04 (0.98, 1.11)		
MSSA	**1.12 (1.10, 1.14)**	1.00 (0.97, 1.03)		
MRSA	0.98 (0.95, 1.02)	**1.07 (1.01, 1.13)**		
DS *P. aeruginosa*	0.99 (0.96, 1.02)	1.01 (0.96, 1.07)		
DR *P. aeruginosa*	0.98 (0.93, 1.03)	1.02 (0.95, 1.10)		
Elixhauser index	**1.01 (1.00, 1.01)**	1.00 (1.00, 1.01)		
	Environmental flora			
	**DS *P. aeruginosa***	**DR *P. aeruginosa***		
Colonization pressure				
DS Enterobacterales	**0.99 (0.97, 1.00)**	0.98 (0.96, 1.00)		
ESBL Enterobacterales	**0.97 (0.94, 1.00)**	1.00 (0.94, 1.05)		
C. difficile	0.96 (0.91, 1.01)	1.02 (0.93, 1.12)		
VSE	1.03 (0.99, 1.06)	0.95 (0.89, 1.02)		
VRE	**0.94 (0.89, 1.00)**	**1.11 (1.00, 1.23)**		
MSSA	1.02 (0.99, 1.05)	0.98 (0.93, 1.05)		
MRSA	1.00 (0.95, 1.05)	1.07 (0.98, 1.17)		
DS *P. aeruginosa*	**1.10 (1.05, 1.15)**	0.94 (0.86, 1.04)		
DR *P. aeruginosa*	0.99 (0.93, 1.06)	**1.29 (1.14, 1.45)**		
Elixhauser index	**1.01 (1.00, 1.01)**	1.01 (1.00, 1.02)		

Table S8: Coefficients and 95% CIs for conditional logistic regression models estimating the impact of colonization pressure on the odds of hospital acquisition.

Coefficients in red and bold indicate estimate reached statistical significance

Target pathogen	AUROC (95% CI)	AUPRC (95% CI)	PPV	NPV	LR+	LR-
Conditional logistic regression						
E. coli	0.56 (0.55, 0.56)	0.42 (0.42, 0.42)	0.40	0.66	1.14	0.86
ESBL *E. coli*	0.59 (0.57, 0.61)	0.43 (0.41,0.45)	0.40	0.71	1.26	0.77
K. pneumoniae	0.57 (0.56, 0.57)	0.30 (0.30, 0.31)	0.30	0.78	1.21	0.81
ESBL *K. pneumoniae*	0.66 (0.63, 0.69)	0.58 (0.56, 0.61)	0.51	0.68	1.49	0.65
C. difficile	0.66 (0.66, 0.66)	0.50 (0.50, 0.51)	0.47	0.74	1.52	0.61
VS *E. faecalis*	0.61 (0.61, 0.62)	0.41 (0.40, 0.41)	0.39	0.75	1.38	0.70
VR *E. faecium*	0.65 (0.64, 0.66)	0.46 (0.44, 0.48)	0.45	0.77	1.60	0.58
MSSA	0.63 (0.62, 0.63)	0.42 (0.41, 0.43)	0.39	0.77	1.40	0.67
MRSA	0.57 (0.56, 0.58)	0.34 (0.34, 0.36)	0.33	0.76	1.21	0.79
DS P. aeruginosa	0.57 (0.56, 0.58)	0.39 (0.39, 0.40)	0.37	0.71	1.20	0.82
DR *P. aeruginosa*	0.64 (0.62, 0.66)	0.41 (0.39, 0.43)	0.39	0.77	1.45	0.67
XGBoost						
E. coli	0.57 (0.56, 0.58)	0.43 (0.40, 0.45)	0.40	0.68	1.13	0.82
ESBL *E. coli*	0.57 (0.55, 0.59)	0.35 (0.25, 0.45)	0.35	0.70	1.15	0.92
K. pneumoniae	0.56 (0.54, 0.58)	0.38 (0.33, 0.42)	0.40	0.69	1.29	0.87
ESBL *K. pneumoniae*	0.56 (0.49, 0.62)	0.46 (0.18, 0.74)	0.42	0.71	1.35	0.75
C. difficile	0.63 (0.60, 0.67)	0.52 (0.46, 0.57)	0.50	0.70	1.54	0.68
VS *E. faecalis*	0.59 (0.57, 0.61)	0.46 (0.41, 0.50)	0.43	0.70	1.33	0.75
VR *E. faecium*	0.59 (0.56, 0.61)	0.47 (0.24, 0.70)	0.50	0.62	1.35	0.83
MSSA	0.62 (0.60, 0.65)	0.45 (0.38, 0.52)	0.40	0.75	1.50	0.76
MRSA	0.57(0.54, 0.60)	0.37 (0.29, 0.45)	0.33	0.69	1.05	0.97
DS P. aeruginosa	0.57 (0.55, 0.59)	0.38 (0.33, 0.42)	0.40	0.69	1.29	0.87
DR *P. aeruginosa*	0.62 (0.58, 0.67)	0.49 (0.31, 0.67)	0.41	0.61	1.06	0.97

Table S9: Performance of conditional logistic regression and XGBoost models.

Abbreviations, AUROC, area under the receiver operator curve; AUPRC, area under the precision-recall curve; PPV, positive predictive value; NPV, negative predictive value; LR+, likelihood ratio positive; LR- likelihood ratio negative.

Model feature	Target organism			
Gram negative enteric flora				
	E. coli	**ESBL *E. coli***	***K. pneumoniae***	**ESBL *K. pneumoniae***
Colonization pressure				
DS Enterobacterales	0.020 +/- 0.000	0.011 +/- 0.000	0.022 +/- 0.000	0.005 +/- 0.000
ESBL Enterobacterales	0.015 +/- 0.000	0.016 +/- 0.000	0.013 +/- 0.000	0.004 +/- 0.000
C. difficile	0.011 +/- 0.000	0.009 +/- 0.000	0.013 +/- 0.000	0.000 +/- 0.000
VSE	0.009 +/- 0.000	0.014 +/- 0.000	0.021 +/- 0.000	0.000 +/- 0.000
VRE	0.009 +/- 0.000	0.008 +/- 0.000	0.017 +/- 0.000	0.000 +/- 0.000
MSSA	0.016 +/- 0.000	0.008 +/- 0.000	0.017 +/- 0.000	0.001 +/- 0.000
MRSA	0.015 +/- 0.000	0.012 +/- 0.000	0.032 +/- 0.000	0.000 +/- 0.000
DS *P. aeruginosa*	0.010 +/- 0.000	0.003 +/- 0.000	0.011 +/- 0.000	0.000 +/- 0.000
DR *P. aeruginosa*	0.008 +/- 0.000	0.007 +/- 0.000	0.014 +/- 0.000	0.000 +/- 0.000
Elixhauser index	0.007 +/- 0.000	0.014 +/- 0.000	0.036 +/- 0.000	0.000 +/- 0.000
Gram positive enteric flora				
	C. difficile	**VS *E. faecalis***	**VR *E. faecium***	
Colonization pressure				
DS Enterobacterales	0.005 +/- 0.000	0.042 +/- 0.001	0.068 +/- 0.002	
ESBL Enterobacterales	0.005 +/- 0.000	0.018 +/- 0.000	0.096 +/- 0.003	
C. difficile	0.022+/- 0.000	0.036 +/- 0.001	0.037 +/- 0.001	
VSE	0.009 +/- 0.000	0.091 +/- 0.001	0.073 +/- 0.002	
VRE	0.003 +/- 0.000	0.021 +/- 0.000	0.073 +/- 0.002	
MSSA	0.007 +/- 0.001	0.033 +/- 0.000	0.034 +/- 0.001	
MRSA	0.014 +/- 0.000	0.018 +/- 0.000	0.064 +/- 0.002	
DS *P. aeruginosa*	0.015+/- 0.000	0.018 +/- 0.000	0.048 +/- 0.001	
DR *P. aeruginosa*	0.010 +/- 0.000	0.030 +/- 0.000	0.028 +/- 0.001	
Elixhauser index	0.010 +/- 0.000	0.013 +/- 0.000	0.033 +/- 0.001	
Skin flora				
	**MSSA**	**MRSA**		
Colonization pressure				
DS Enterobacterales	0.085 +/- 0.000	0.003 +/- 0.001		
ESBL Enterobacterales	0.064 +/- 0.001	0.001 +/- 0.000		
C. difficile	0.029 +/- 0.000	0.000 +/- 0.000		
VSE	0.047 +/- 0.000	0.002 +/- 0.000		
VRE	0.015 +/- 0.000	0.002 +/- 0.000		
MSSA	0.086 +/- 0.001	0.000 +/- 0.000		
MRSA	0.017 +/- 0.000	0.000 +/- 0.000		
DS *P. aeruginosa*	0.028 +/- 0.000	0.002 +/- 0.000		
DR *P. aeruginosa*	0.013 +/- 0.000	0.003 +/- 0.000		
Elixhauser index	0.056 +/- 0.000	0.004 +/- 0.000		
Environmental flora				
	**DS *P. aeruginosa***	**DR *P. aeruginosa***		
Colonization pressure				
DS Enterobacterales	0.019 +/- 0.000	0.008 +/- 0.000		
ESBL Enterobacterales	0.014 +/- 0.000	0.007 +/- 0.000		
C. difficile	0.020 +/- 0.000	0.008 +/- 0.000		
VSE	0.010 +/- 0.000	0.012 +/- 0.000		
VRE	0.009 +/- 0.000	0.008 +/- 0.000		
MSSA	0.008 +/- 0.000	0.006 +/- 0.000		
MRSA	0.014 +/- 0.000	0.013 +/- 0.000		
DS *P. aeruginosa*	0.031 +/- 0.000	0.016 +/- 0.000		
DR *P. aeruginosa*	0.009 +/- 0.000	0.022 +/- 0.000		
Elixhauser index	0.013 +/- 0.000	0.013 +/- 0.000		

Table S10: Mean SHAP values for XGBoost models.

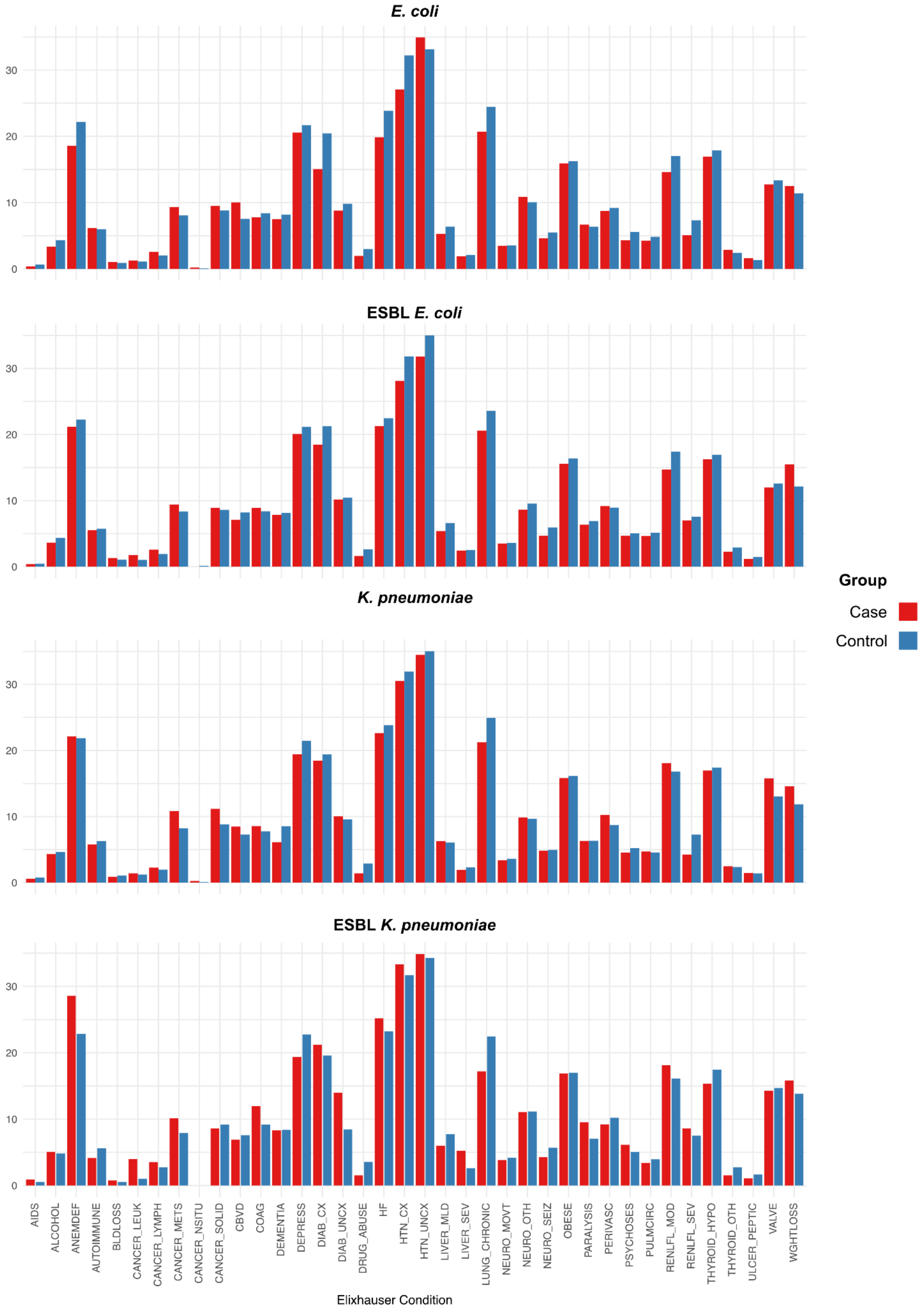

Figure S1: Distribution of individual elixhauser categories for drug susceptible and ESBL *E. coli* and *K. pneumoniae*.

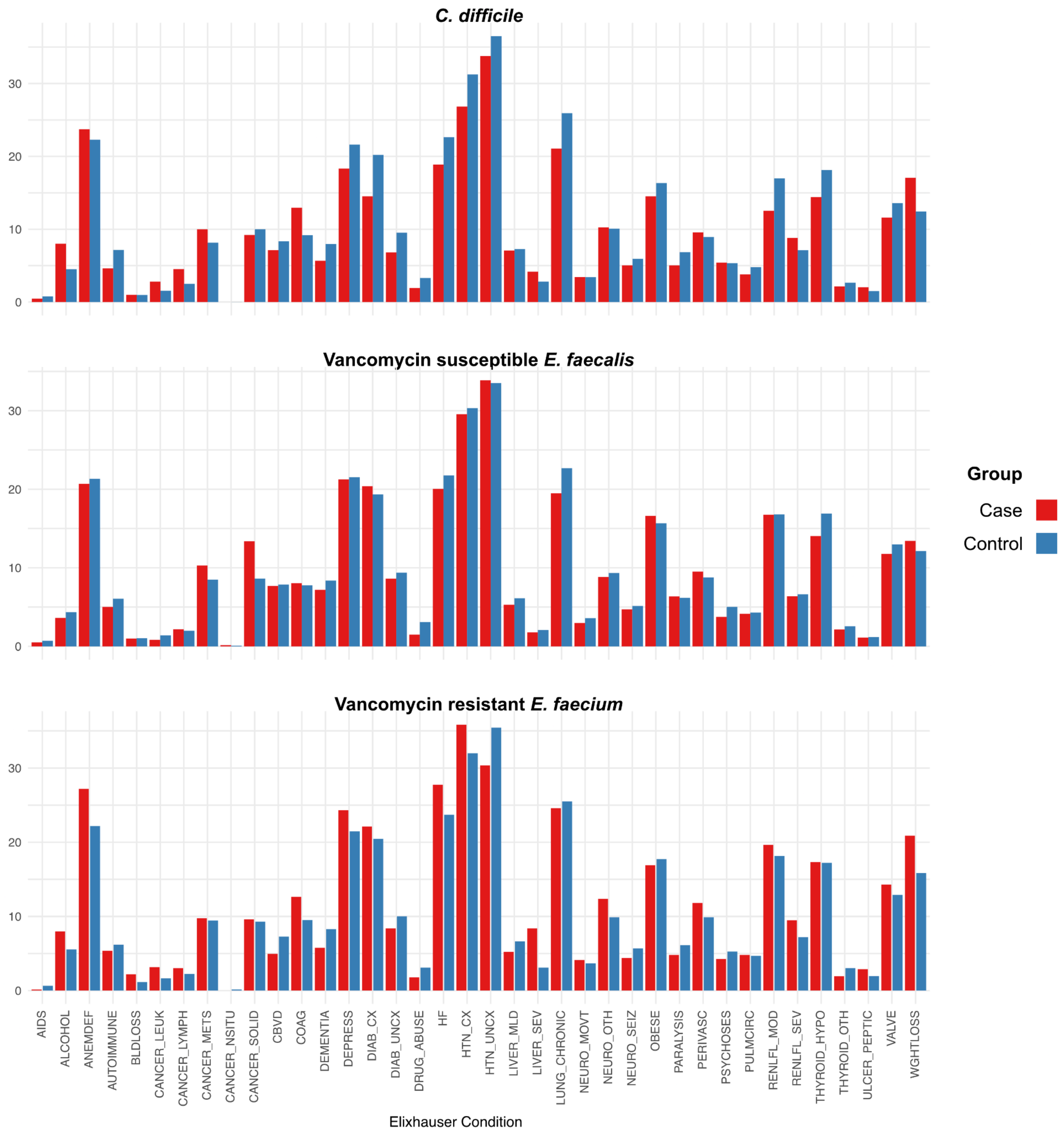

Figure S2: Distribution of individual elixhauser categories for *C. difficile,* vancomycin susceptible *E. faecalis*, and vancomycin resistant *E. faecium.*

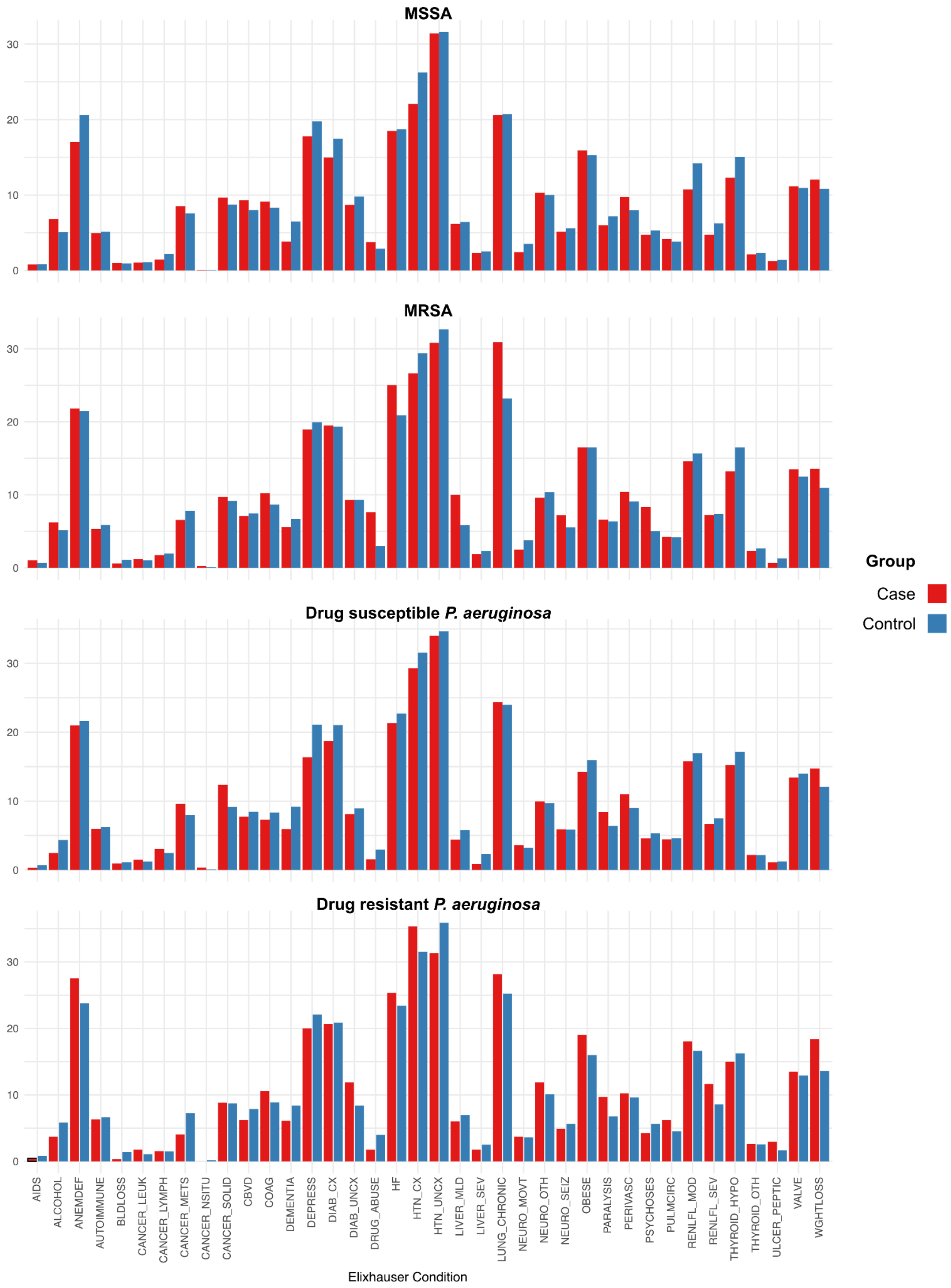

Figure S3: Distribution of individual elixhauser categories for MSSA, MRSA, drug susceptible and drug resistant *P. aeruginosa.*

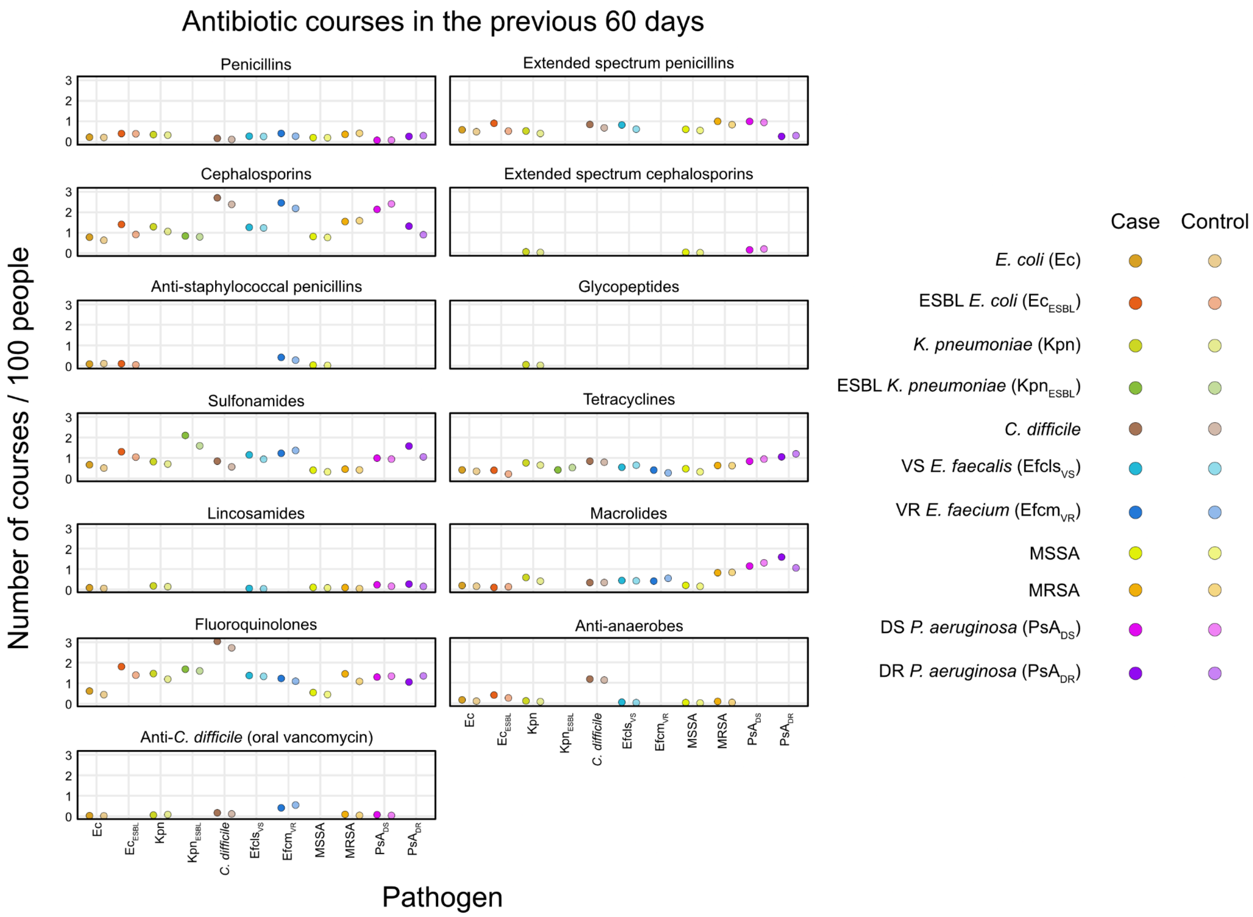

Figure S4: Number of antibiotic courses per 100 people in the previous 60 days by class.

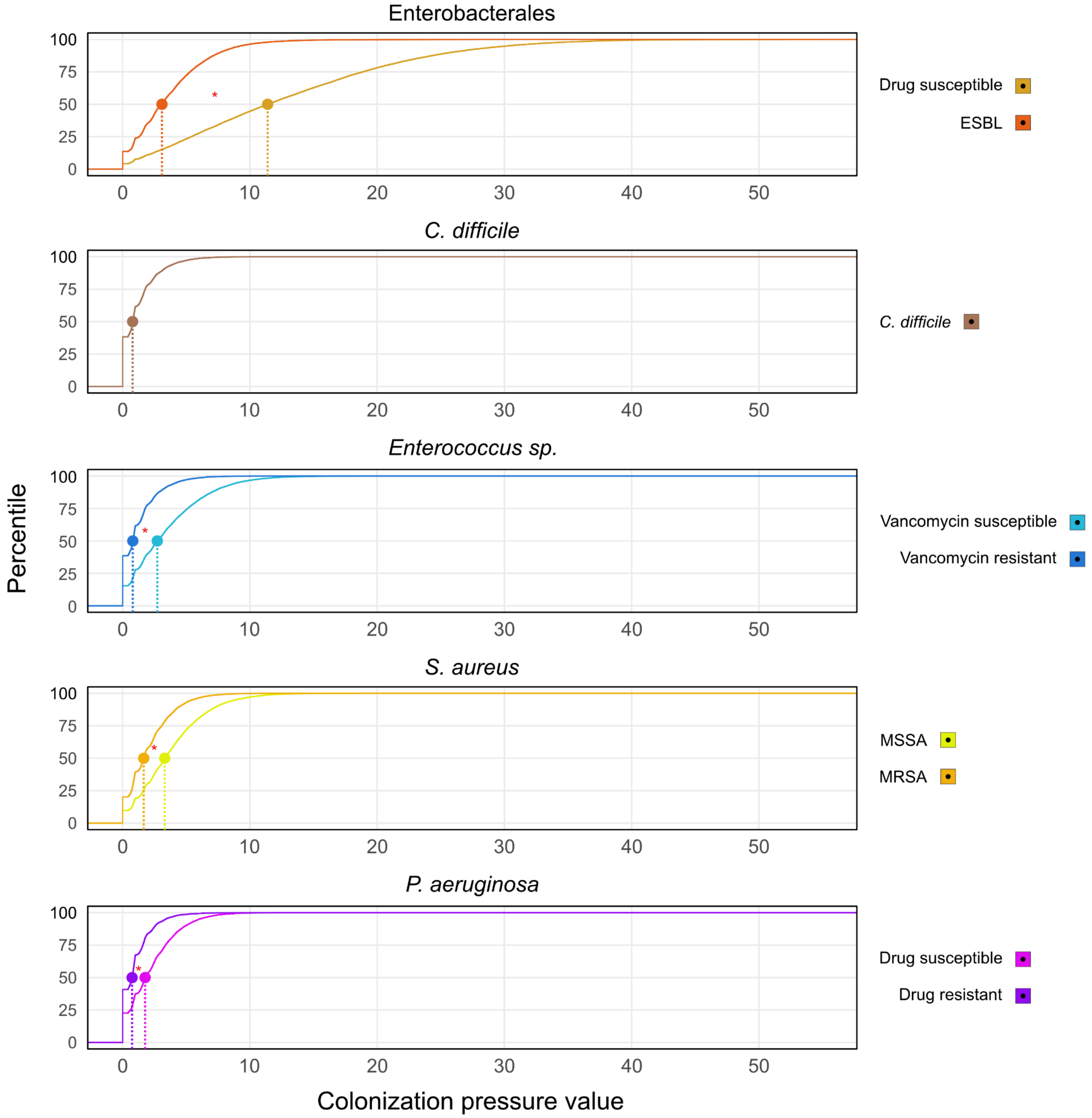

Figure S5: Cumulative distributions of colonization pressure. Vertical line indicates the median value and the red asterisk indicates p < 0.05 for comparison of distributions.

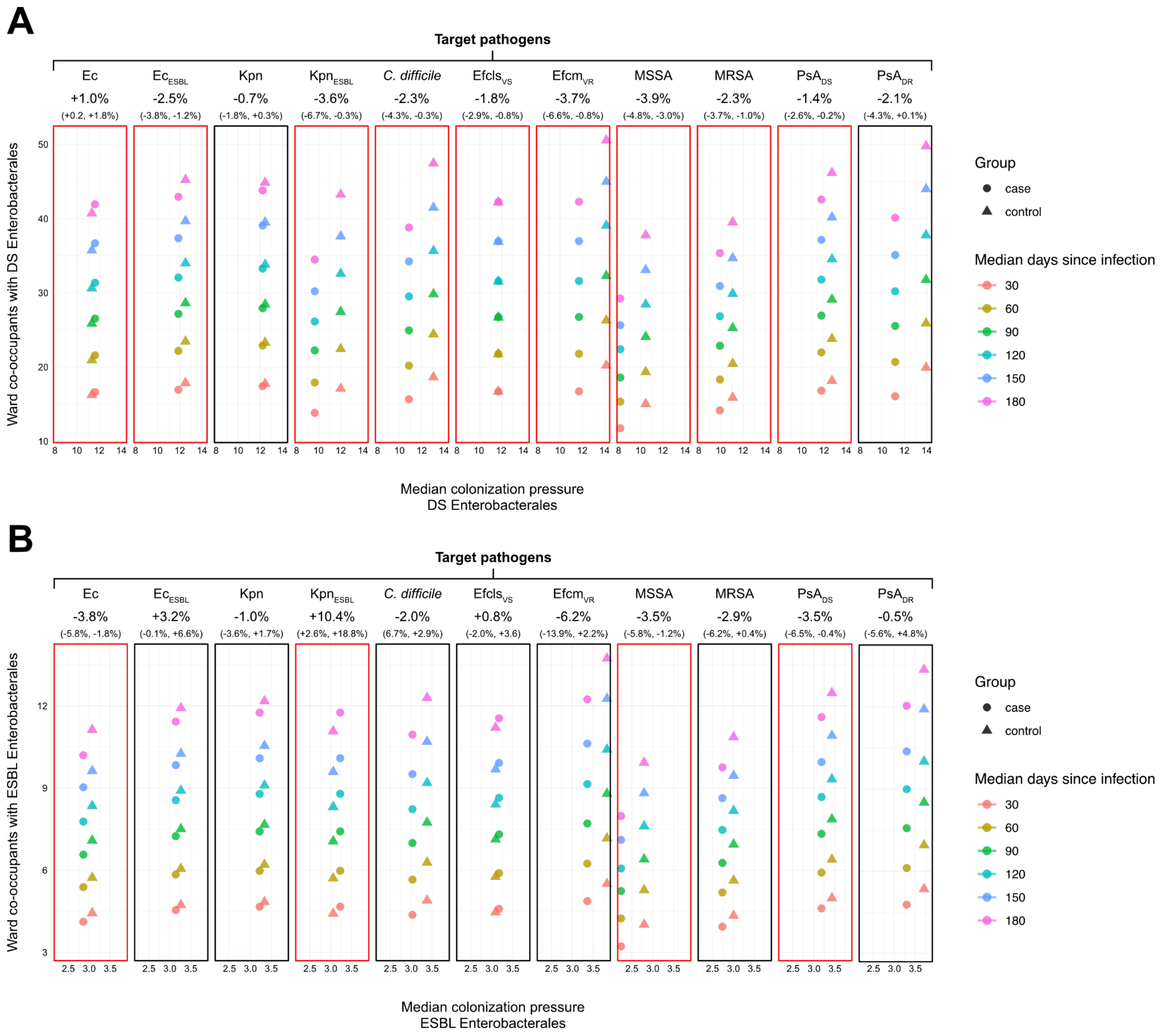

Figure S6: Number of ward co-occupants with A) drug susceptible Enterobacterales and B) ESBL Enterobacterales by target pathogen cohort. X-axis values represent median colonization pressure observed for cases and controls within the target pathogen cohort. Estimates based on relationship shown on Figure S6, and stratified by the median number of days since ward co-occupants' last culture was positive for the colonization pressure organism (or organism set). Conditional logistic regression model coefficients shown above panel. Panel/s highlighted in red indicate statistically significant model coefficients.

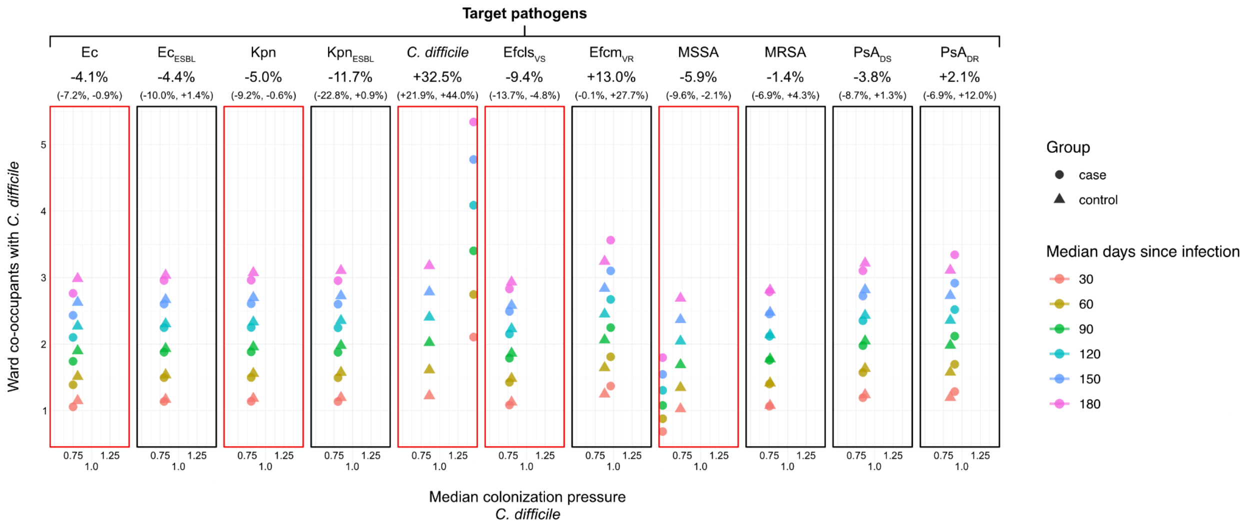

Figure S7: Number of ward co-occupants with *C. difficile* by target pathogen cohort. X-axis values represent median colonization pressure observed for cases and controls within the target pathogen cohort. Estimates based on relationship shown on Figure S6, and stratified by the median number of days since ward co-occupants' last culture was positive for the colonization pressure organism (or organism set). Conditional logistic regression model coefficients shown above panel. Panel/s highlighted in red indicate statistically significant model coefficients.

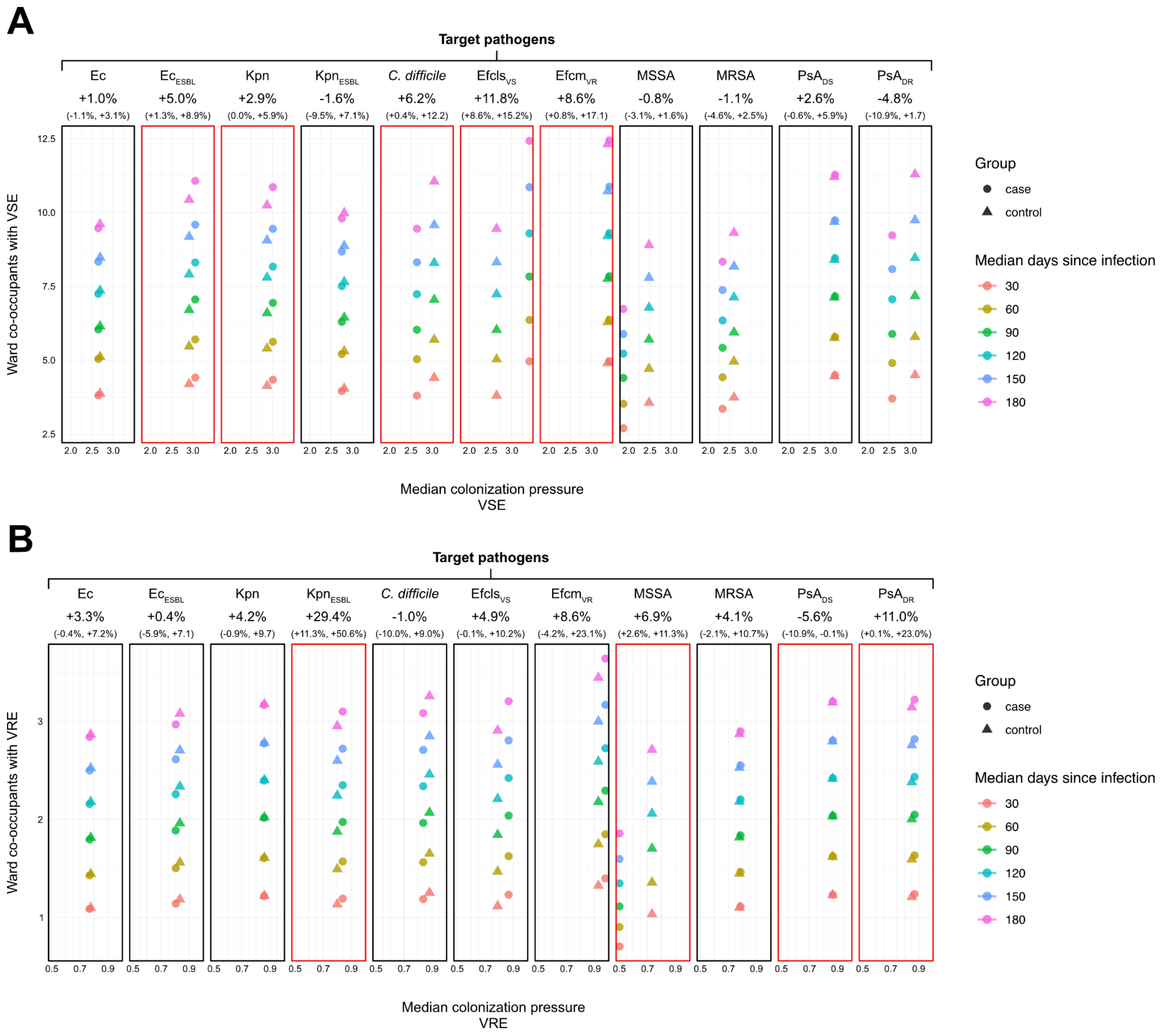

Figure S8: Number of ward co-occupants with A) vancomycin susceptible and B) vancomycin resistant *Enterococcus* species by target pathogen cohort. X-axis values represent median colonization pressure observed for cases and controls within the target pathogen cohort. Estimates based on relationship shown on Figure S6, and stratified by the median number of days since ward co-occupants' last culture was positive for the colonization pressure organism (or organism set). Conditional logistic regression model coefficients shown above panel. Panel/s highlighted in red indicate statistically significant model coefficients.

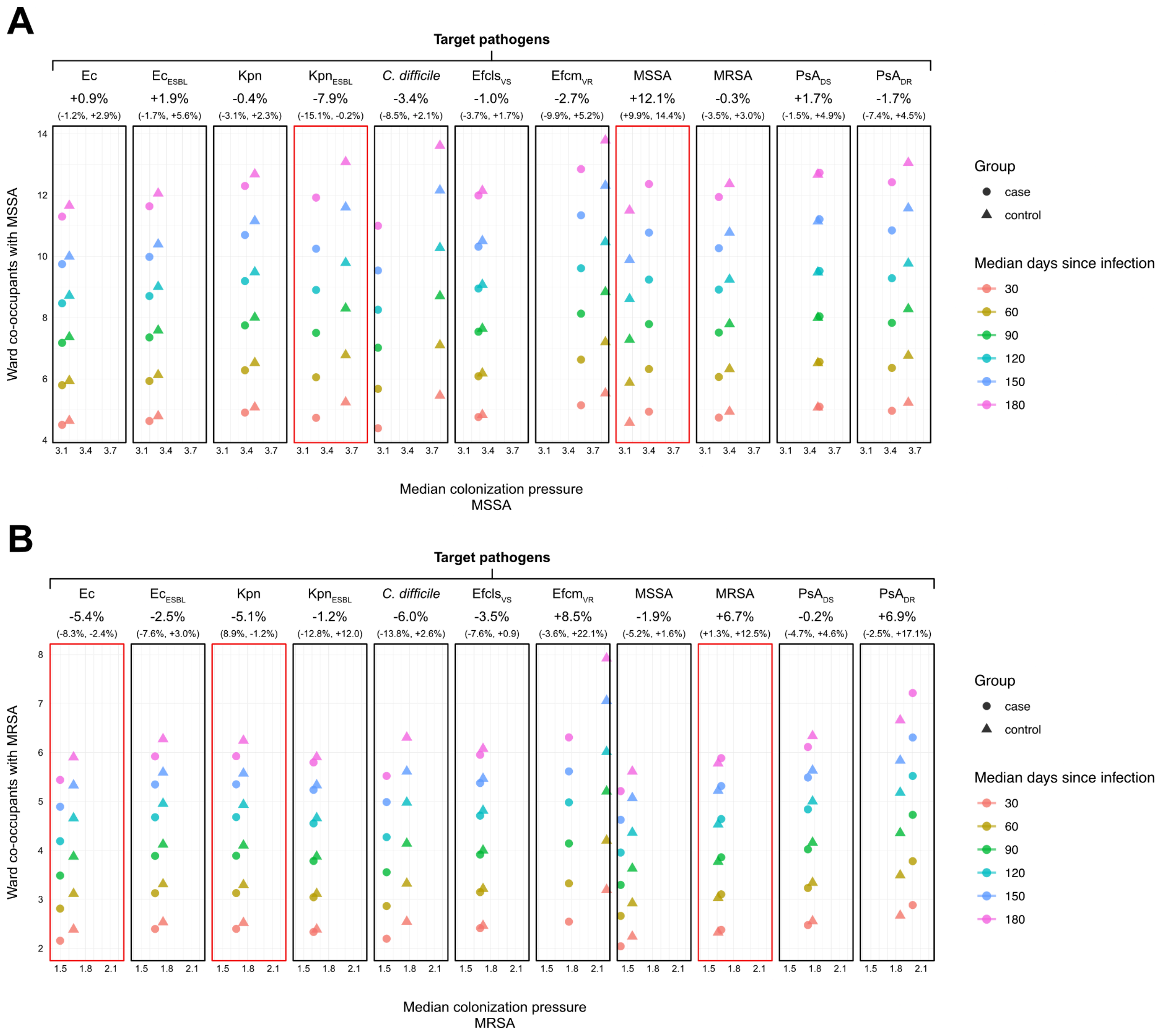

Figure S9: Number of ward co-occupants with A) MSSA and B) MRSA by target pathogen cohort. X-axis values represent median colonization pressure observed for cases and controls within the target pathogen cohort. Estimates based on relationship shown on Figure S6, and stratified by the median number of days since ward co-occupants' last culture was positive for the colonization pressure organism (or organism set). Conditional logistic regression model coefficients shown above panel. Panel/s highlighted in red indicate statistically significant model coefficients.

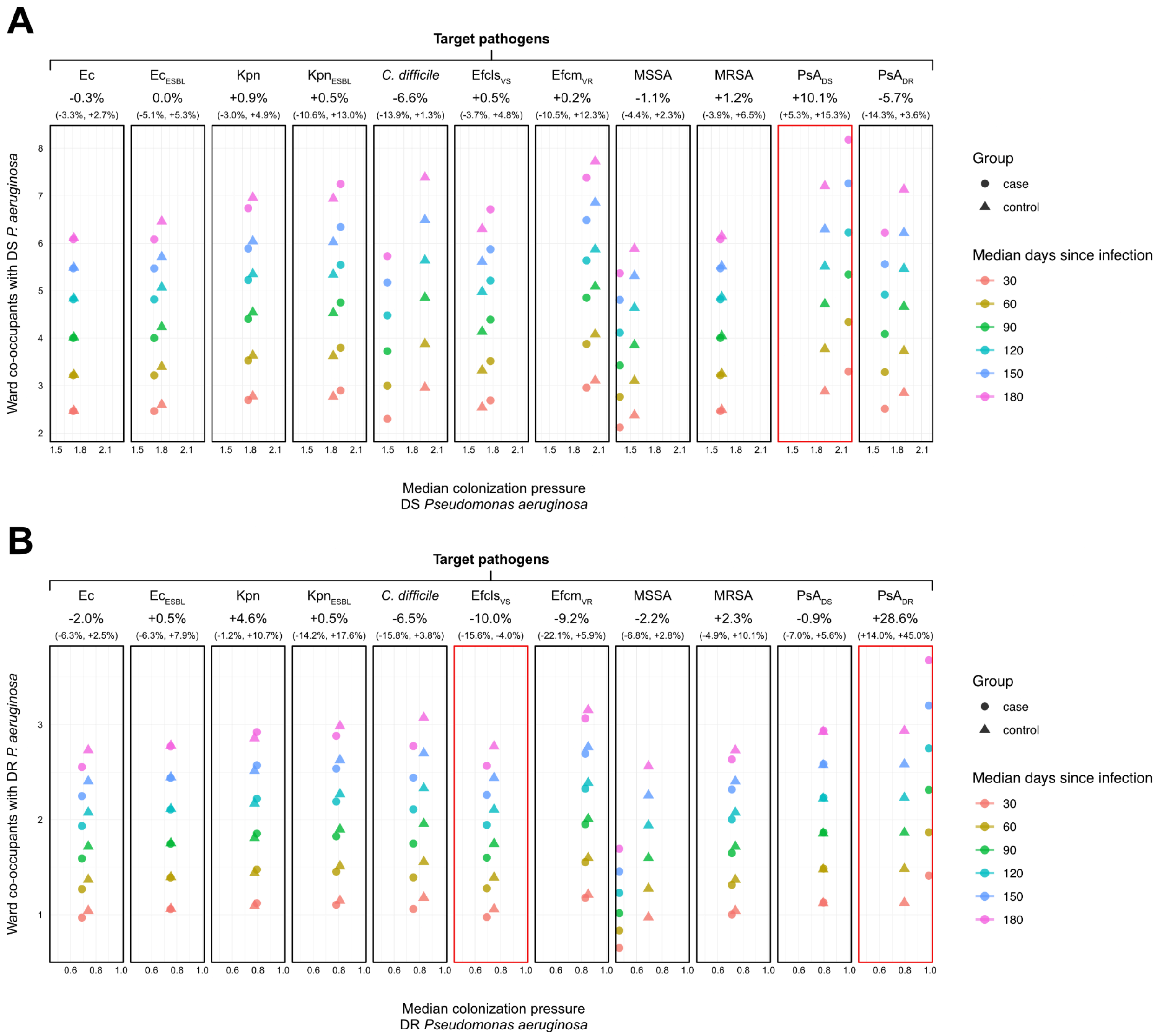

Figure S10: Number of ward co-occupants with A) drug susceptible *P. aeruginosa* and B) drug resistant *P. aeruginosa* by target pathogen cohort. X-axis values represent median colonization pressure observed for cases and controls within the target pathogen cohort. Estimates based on relationship shown on Figure S6, and stratified by the median number of days since ward co-occupants' last culture was positive for the colonization pressure organism (or organism set). Conditional logistic regression model coefficients shown above panel. Panel/s highlighted in red indicate statistically significant model coefficients.

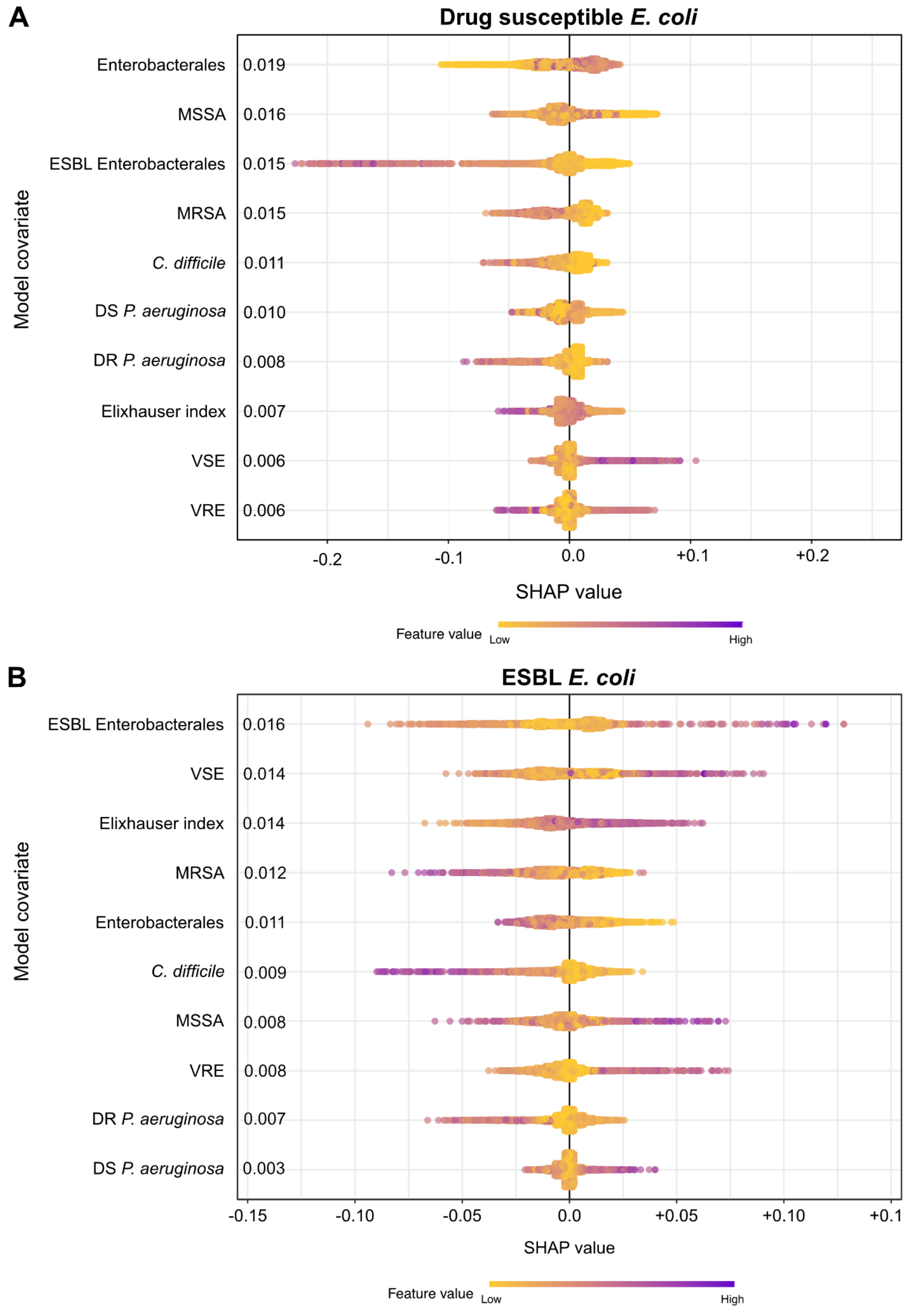

Figure S11: SHAP values for XGB models predicting nosocomial acquisition of A) drug susceptible and B) ESBL *E. coli*. Mean SHAP value on left of plot. All features represent organism-specific colonization pressure except for Elixhauser index. Features are ranked by importance.

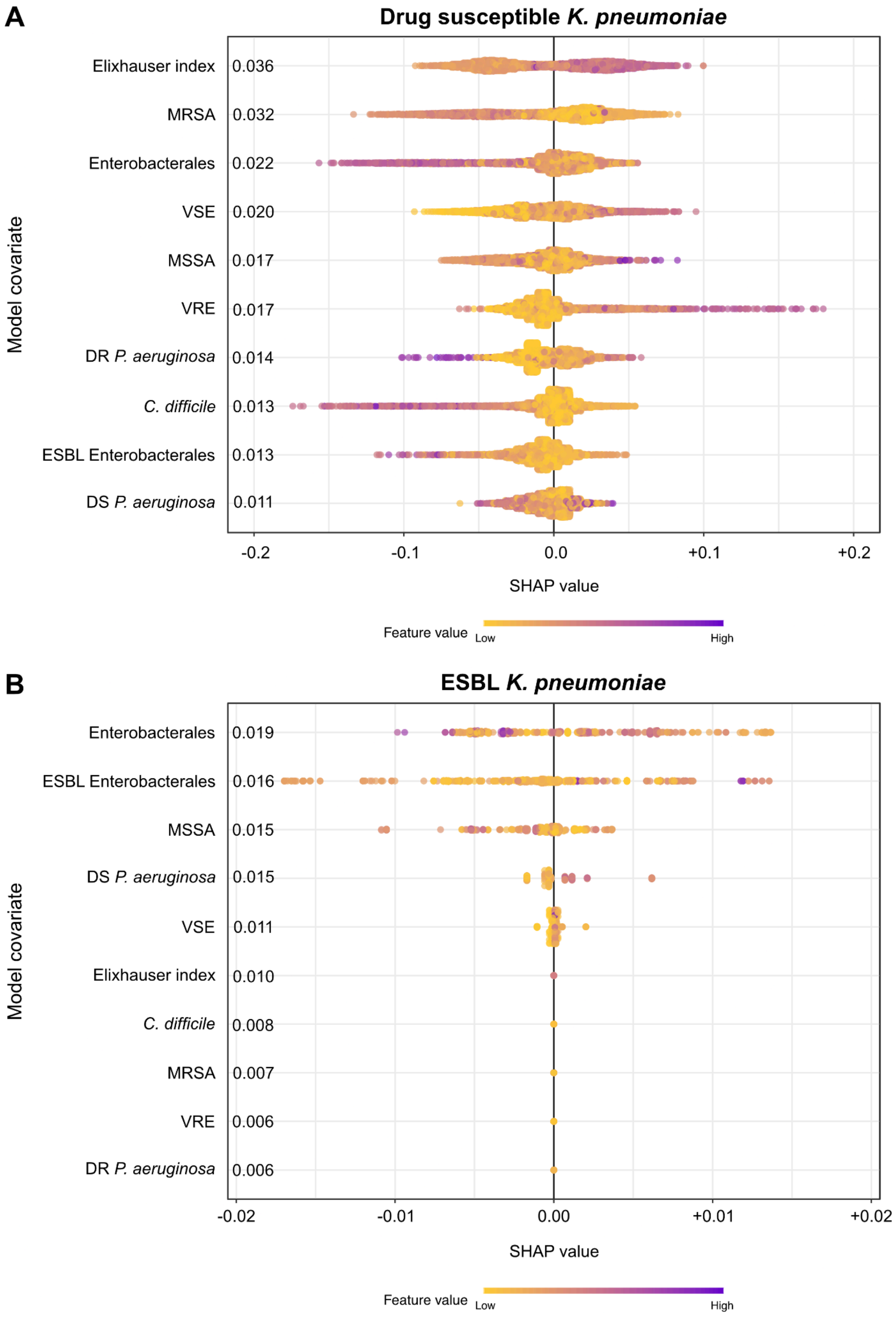

Figure S12: SHAP values for XGB models predicting nosocomial acquisition of A) drug susceptible and B) ESBL *K.pneumoniae*. Mean SHAP value on left of plot. All features represent organism-specific colonization pressure except for Elixhauser index. Features are ranked by importance.

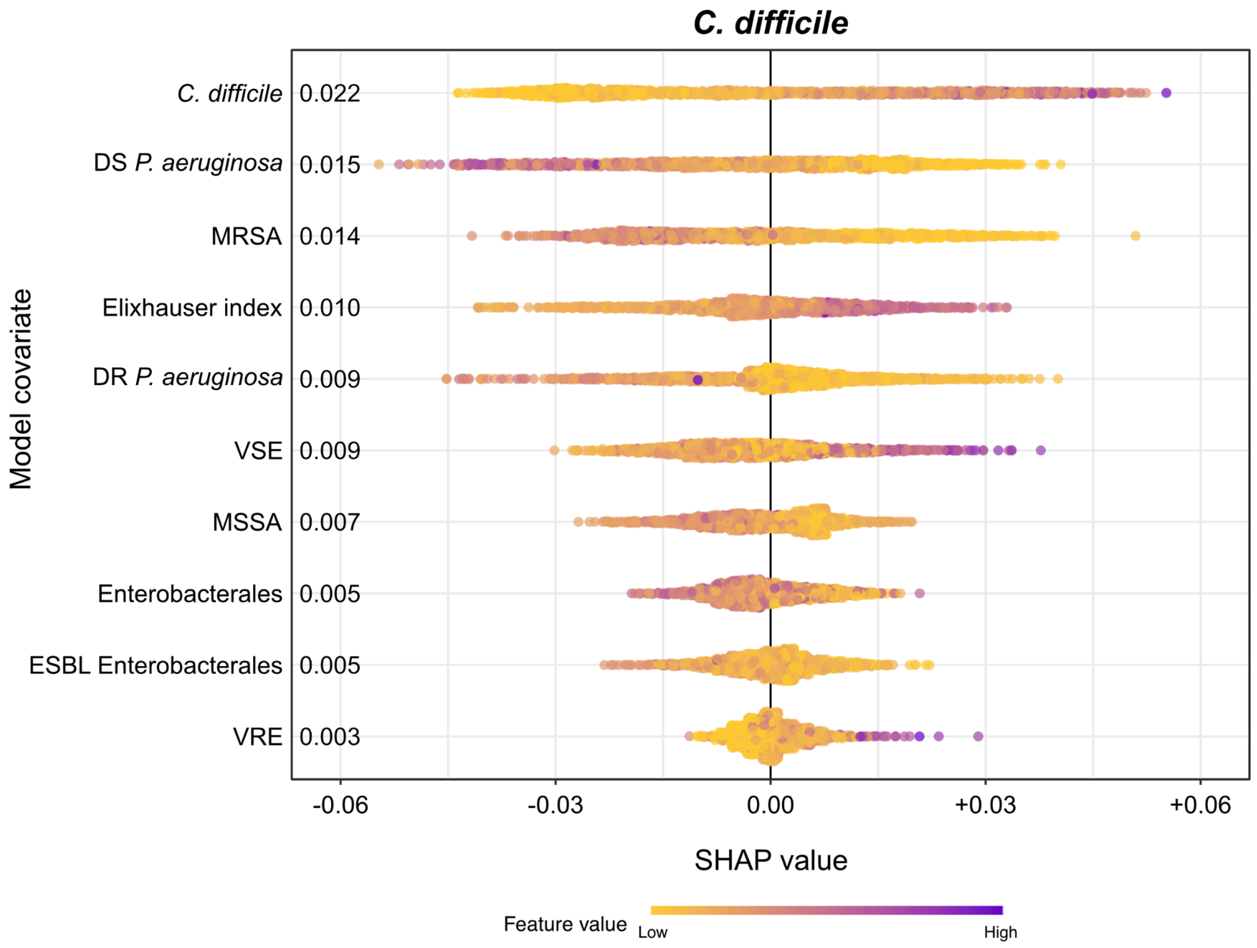

Figure S13: SHAP values for XGB models predicting nosocomial acquisition of *C. difficile*. Mean SHAP value on left of plot. All features represent organism-specific colonization pressure except for Elixhauser index. Features are ranked by importance.

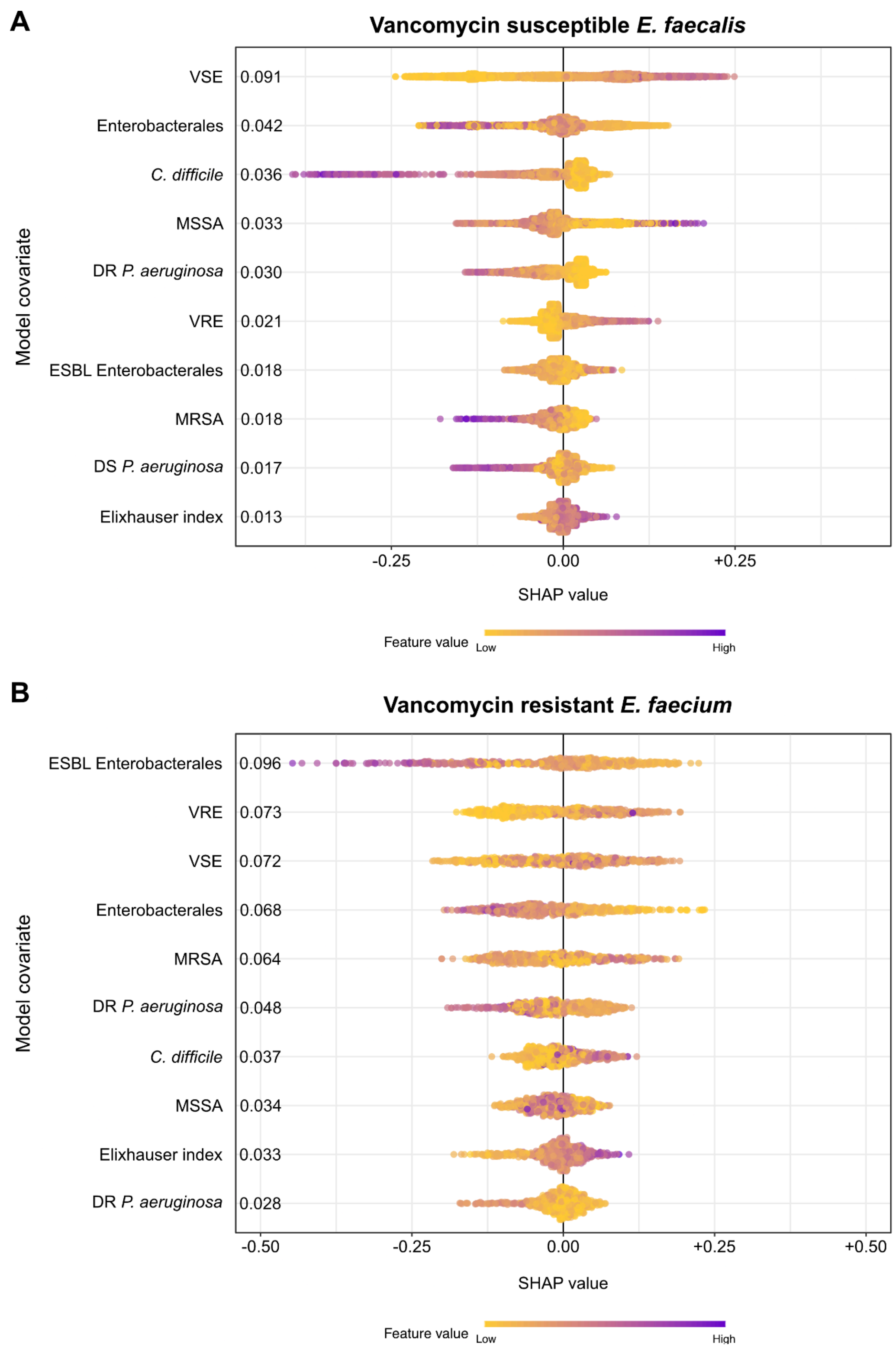

Figure S14: SHAP values for A) vancomycin susceptible *E. faecalis* and B) vancomycin resistant *E. faecium*. Mean SHAP value on left of plot. All features represent organism-specific colonization pressure except for Elixhauser index. Features are ranked by importance.

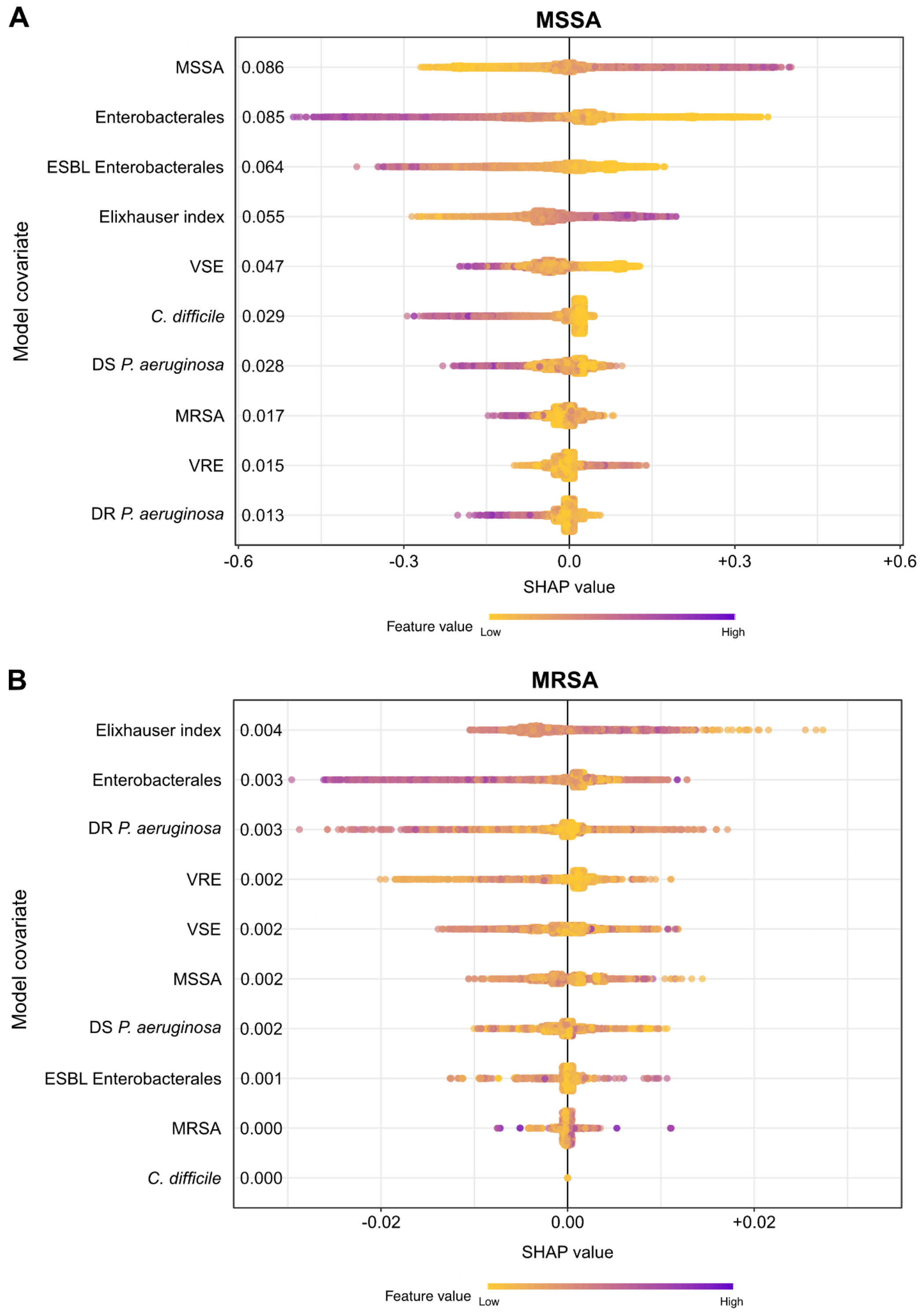

Figure S15: SHAP values for XGB models predicting nosocomial acquisition of A) MSSA and B) MRSA. Mean SHAP value on left of plot. All features represent organism-specific colonization pressure except for Elixhauser index. Features are ranked by importance.

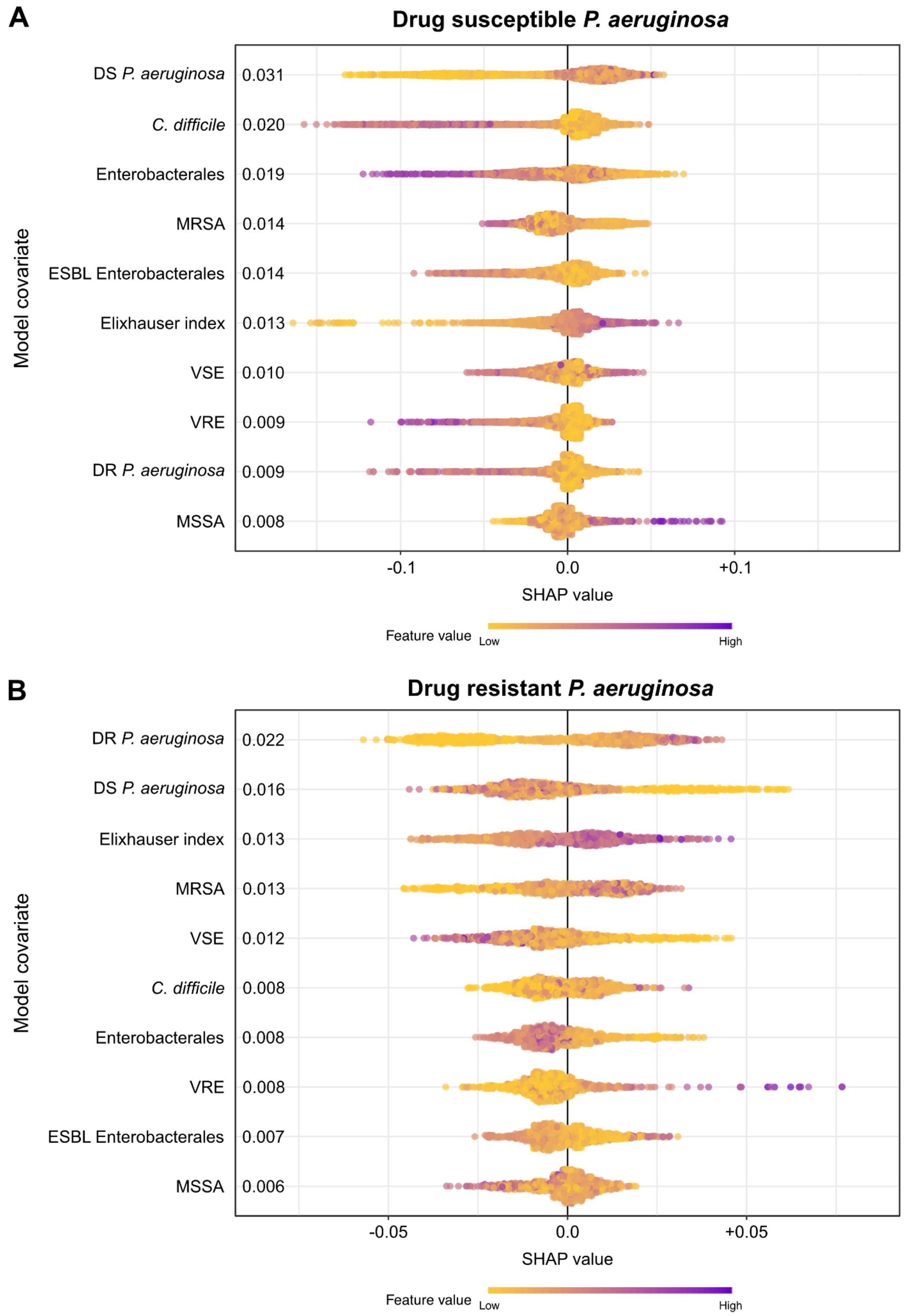

Figure S16: SHAP values for XGB models predicting nosocomial acquisition of A) drug susceptible and B) drug resistant *P. aeruginosa*. Mean SHAP value on left of plot. All features represent organism-specific colonization pressure except for Elixhauser index. Features are ranked by importance.
