# Supplementary material for: Using electronic health records to assess the relationship between colonization pressure and nosocomial pathogen acquisition
Source: Nat Commun. 2026 Feb 24;17:3134. doi: 10.1038/s41467-026-69873-4 (PMC13043742; doi:10.1038/s41467-026-69873-4)
Supplement: Supplementary file 2 — Reporting Summary [file 41467_2026_69873_MOESM2_ESM.pdf]

nature portfolio

Corresponding author(s): S Kanjilal

Last updated by author(s): Feb 5, 2026

Reporting Summary

Nature Portfolio wishes to improve the reproducibility of the work that we publish. This form provides structure for consistency and transparency in reporting. For further information on Nature Portfolio policies, see our Editorial Policies and the Editorial Policy Checklist.

Statistics

For all statistical analyses, confirm that the following items are present in the figure legend, table legend, main text, or Methods section.

n/a	Confirmed	
☐	☒	The exact sample size (*n*) for each experimental group/condition, given as a discrete number and unit of measurement
☐	☒	A statement on whether measurements were taken from distinct samples or whether the same sample was measured repeatedly
☐	☒	The statistical test(s) used AND whether they are one- or two-sided<br>*Only common tests should be described solely by name; describe more complex techniques in the Methods section.*
☐	☒	A description of all covariates tested
☒	☐	A description of any assumptions or corrections, such as tests of normality and adjustment for multiple comparisons
☐	☒	A full description of the statistical parameters including central tendency (e.g. means) or other basic estimates (e.g. regression coefficient) AND variation (e.g. standard deviation) or associated estimates of uncertainty (e.g. confidence intervals)
☐	☒	For null hypothesis testing, the test statistic (e.g. *F*, *t*, *r*) with confidence intervals, effect sizes, degrees of freedom and *P* value noted<br>*Give P values as exact values whenever suitable.*
☒	☐	For Bayesian analysis, information on the choice of priors and Markov chain Monte Carlo settings
☒	☐	For hierarchical and complex designs, identification of the appropriate level for tests and full reporting of outcomes
☒	☐	Estimates of effect sizes (e.g. Cohen's *d*, Pearson's *r*), indicating how they were calculated

Our web collection on statistics for biologists contains articles on many of the points above.

Software and code

Policy information about availability of computer code

Data collection	Data were extracted from the Mass General Brigham electronic health record (EHR) data warehouse using custom SQL scripts.
Data analysis	All analyses were conducted using R (version 4.4.0) and Python (version 3.6.15). Conditional logistic regression was implemented using the survival R package. XGBoost models were implemented using the xgboost Python package. SHAP values were computed using the shap Python library. The full analytic pipeline, including preprocessing and modeling code, is available at https://github.com/sanjatkanjilal/nosocomial-acquisition_colonization-pressure.

For manuscripts utilizing custom algorithms or software that are central to the research but not yet described in published literature, software must be made available to editors and reviewers. We strongly encourage code deposition in a community repository (e.g. GitHub). See the Nature Portfolio guidelines for submitting code & software for further information.

Data

Policy information about availability of data

All manuscripts must include a data availability statement. This statement should provide the following information, where applicable:

- Accession codes, unique identifiers, or web links for publicly available datasets
- A description of any restrictions on data availability
- For clinical datasets or third party data, please ensure that the statement adheres to our policy

A de-identified version of the final cleaned dataset used for the colonization pressure models is available on PhysioNet under the project title “Predictors of hospital

onset infection: A matched retrospective cohort dataset" (https://doi.org/10.13026/k70x-0m81). Access is provided through PhysioNet's credentialed access process and requires signing a data use agreement. The original raw data from the Mass General Brigham electronic health record system are not publicly available due to patient privacy and institutional restrictions.

Research involving human participants, their data, or biological material

Policy information about studies with human participants or human data. See also policy information about sex, gender (identity/presentation), and sexual orientation and race, ethnicity and racism.

Reporting on sex and gender	Sex (biological attribute) and gender were not explicitly considered in the study design. Matching for controls included age and sex, with sex determined from the electronic health record. No sex- or gender-stratified analyses were performed. Individual-level sex-disaggregated data were not shared in the public dataset.
Reporting on race, ethnicity, or other socially relevant groupings	Race, ethnicity, and other socially relevant variables were not used in matching or modeling and were not collected or analyzed in this study. Therefore, no confounding adjustment was performed for these variables.
Population characteristics	The study population included adults (≥18 years) admitted to any of the 10 hospitals within the Mass General Brigham system between May 25, 2015 and July 7, 2024. Cases had the organism of interest in a clinical or surveillance culture at any point between day 3 and day 30 after entry into their index room. Controls were matched to cases based on age, sex, length of stay in the room (or time to infection), history of prior surgery in the previous 90 days, and prior antibiotic exposure (stratified by 14 different classes of antibiotics).
Recruitment	Participants were not prospectively recruited. This was a retrospective observational study using existing EHR data. Inclusion criteria required patients (both cases and controls) to have stayed in a single room for ≥48 hours, excluding the emergency room, with no prior evidence of the target organism and no recent antibiotic use, as detailed in the Methods section.
Ethics oversight	This study was deemed exempt by the Institutional Review Board of Mass General Brigham.

Note that full information on the approval of the study protocol must also be provided in the manuscript.

Field-specific reporting

Please select the one below that is the best fit for your research. If you are not sure, read the appropriate sections before making your selection.

☒ Life sciences ☐ Behavioural & social sciences ☐ Ecological, evolutionary & environmental sciences

For a reference copy of the document with all sections, see nature.com/documents/nr-reporting-summary-flat.pdf

Life sciences study design

All studies must disclose on these points even when the disclosure is negative.

Sample size	The sample size was determined based on all eligible adult patients from the Mass General Brigham system between May 25, 2015 and July 7, 2024 who met strict inclusion/exclusion criteria. These sample sizes were sufficient to detect statistically significant associations for multiple target organisms, as evidenced by confidence intervals that did not cross the null.
Data exclusions	Patients were excluded if they had: (1) less than 48 hours in a single inpatient room during the observation period, (2) more than one room stay in a 48 hour period during the observation period, (3) prior detection of the target organism within 6 or 12 months, or (4) recent antibiotic use (within 7 days prior to room entry). These criteria were pre-specified to minimize confounding and ensure clarity in defining new nosocomial acquisition events.
Replication	This was a retrospective observational study using real-world EHR data and did not involve experimental replication. Model reproducibility was assessed using 5-fold cross-validation, with performance metrics (e.g., AUROC) reported across folds. All results were consistent and stable across validation folds.
Randomization	Participants were not randomized as this was an observational study. Instead, we matched cases to controls on age, sex, surgery history, length of stay, and prior antibiotic exposure. We further statistically controlled for differences in comorbidity burden using multivariate models.
Blinding	Blinding was not applicable to this retrospective EHR-based study.

Reporting for specific materials, systems and methods

We require information from authors about some types of materials, experimental systems and methods used in many studies. Here, indicate whether each material, system or method listed is relevant to your study. If you are not sure if a list item applies to your research, read the appropriate section before selecting a response.

Materials & experimental systems

n/a	Involved in the study
☒	☐ Antibodies
☒	☐ Eukaryotic cell lines
☒	☐ Palaeontology and archaeology
☒	☐ Animals and other organisms
☐	☒ Clinical data
☒	☐ Dual use research of concern
☒	☐ Plants

Methods

n/a	Involved in the study
☒	☐ ChIP-seq
☒	☐ Flow cytometry
☒	☐ MRI-based neuroimaging

Clinical data

Policy information about clinical studies

All manuscripts should comply with the ICMJE guidelines for publication of clinical research and a completed CONSORT checklist must be included with all submissions.

Clinical trial registration	Not applicable. This study was a retrospective observational study and was not registered as a clinical trial.
Study protocol	No formal clinical trial protocol was required. Study design and inclusion/exclusion criteria are fully described in the Methods section of the manuscript.
Data collection	Data were collected retrospectively from the Mass General Brigham electronic health record system. The study included admissions between May 25, 2015 and July 7, 2024, across 10 hospitals in the New England area.
Outcomes	The primary outcome was the odds of nosocomial acquisition of a target pathogen, given the matched case-control design and after controlling for various colonization pressures and comorbidity burden. Eleven separate models were run for 11 target pathogens.

Plants

Seed stocks	n/a
Novel plant genotypes	n/a
Authentication	n/a
